# Sediment Metagenomes as Time Capsules of Lake Microbiomes

**DOI:** 10.1128/mSphere.00512-20

**Published:** 2020-11-04

**Authors:** Rebecca E. Garner, Irene Gregory-Eaves, David A. Walsh

**Affiliations:** a Department of Biology, Concordia University, Montreal, Quebec, Canada; b Department of Biology, McGill University, Montreal, Quebec, Canada; c Groupe de Recherche Interuniversitaire en Limnologie, Montreal, Quebec, Canada; Clemson University

**Keywords:** paleolimnology, shotgun sequencing, metagenomics, DNA preservation, paleogenomics, bacterioplankton, viruses

## Abstract

The reconstruction of ecological time series from lake sediment archives can retrace the environmental impact of human activities. Molecular genetic approaches in paleolimnology have provided unprecedented access to DNA time series, which record evidence of the microbial ecologies that underlaid historical lake ecosystems. Such studies often rely on single-gene surveys, and consequently, the full diversity of preserved microorganisms remains unexplored. In this study, we probed the diversity archived in contemporary and preindustrial sediments by comparative shotgun metagenomic analysis of surface water and sediment samples from three eastern Canadian lakes. In a strategy that was aimed at disentangling historical DNA from the indigenous sediment background, microbial preservation signals were captured by mapping sequence similarities between sediment metagenome reads and reference surface water metagenome assemblies. We detected preserved *Cyanobacteria*, diverse bacterioplankton, microeukaryotes, and viruses in sediment metagenomes. Among the preserved microorganisms were important groups never before reported in paleolimnological reconstructions, including bacteriophages (*Caudovirales*) and ubiquitous freshwater *Betaproteobacteria* (*Polynucleobacter* and *Limnohabitans*). In contrast, ultramicroscopic *Actinobacteria* (“*Candidatus* Nanopelagicales”) and *Alphaproteobacteria* (*Pelagibacterales*) were apparently not well preserved in sediment metagenomes even though they were numerically dominant in surface water metagenomes. Overall, our study explored a novel application of whole-metagenome shotgun sequencing for discovering the DNA remains of a broad diversity of microorganisms preserved in lake sediments. The recovery of diverse microbial time series supports the taxonomic expansion of microbiome reconstructions and the development of novel microbial paleoindicators.

**IMPORTANCE** Lakes are critical freshwater resources under mounting pressure from climate change and other anthropogenic stressors. The reconstruction of ecological time series from sediment archives with paleolimnological techniques has been shown to be an effective means of understanding how humans are modifying lake ecosystems over extended timescales. In this study, we combined shotgun DNA sequencing with a novel comparative analysis of surface water and sediment metagenomes to expose the diversity of microorganisms preserved in lake sediments. The detection of DNA from a broad diversity of preserved microbes serves to more fully reconstruct historical microbiomes and describe preimpact lake conditions.

## Abstract (precis)

Lakes are critical freshwater resources under mounting pressure from climate change and other anthropogenic stressors. The reconstruction of ecological time series from sediment archives with paleolimnological techniques has been shown to be an effective means of understanding how humans are modifying lake ecosystems over extended timescales. In this study, we combined shotgun DNA sequencing with a novel comparative analysis of surface water and sediment metagenomes to expose the diversity of microorganisms preserved in lake sediments. The detection of DNA from a broad diversity of preserved microbes serves to more fully reconstruct historical microbiomes and describe preimpact lake conditions.

## INTRODUCTION

Lakes support human wellbeing and a rich suite of ecological functions. Relative to the surface area they occupy worldwide, lakes contribute disproportionately to global elemental cycles (1, 2). Lakes are also natural repositories of long-term environmental data. Internal lake dynamics and changes driven by terrestrial and atmospheric inputs are integrated and archived through sediment deposition (3, 4). As climate change and other anthropogenic stressors exert increasing pressures on lakes (5), it is of great interest to examine how lakes are being modified from their preindustrial states. In the absence of long-term monitoring, chronologies reconstructed from lake sediment records can describe departure from preimpact baselines (6) and provide insights into the myriad effects of the anthropogenic impact observed since circa 1880 or earlier (7–9). Classical microbial paleoindicators (e.g., diatom and chrysophyte assemblages) identified from subfossils, pigments, and other proxies are widely applied in paleolimnology for inferring key aspects of historical water columns (10, 11).

Molecular genetic techniques have revolutionized environmental microbiology by exposing a vast, uncultured diversity of microorganisms (12). Molecular genetic analysis, advanced by PCR gene amplification and shotgun metagenomics, is continually expanding the body of knowledge on the diversity and importance of microbial communities in all habitable environments, including aquatic ecosystems (13). The development of molecular genetic methods in paleolimnology has enhanced the detection of soft-bodied microorganisms that lack preserved morphological features but that can be tracked in sediment DNA archives (14, 15). The inclusion of a broader diversity of microorganisms in paleolimnological reconstructions allows access to the microbiomes which underlaid lake ecosystems and biogeochemical processes in the past. The detection of preserved microbial diversity further offers opportunities to explore the still largely untapped potential of microbes as paleoindicators, grounded in the ubiquity of microorganisms in aquatic systems, their unparalleled phylogenetic and ecological breadth (16), and their rapid responses to change (17). Microbial time series reconstructed from lake sediment DNA have so far been used to investigate the emergence of toxic and bloom-forming *Cyanobacteria* (18–21), microeukaryotic community succession in response to eutrophication and climate change (22, 23), microbial evolution in response to anthropogenic mercury loading (24), and past methane oxidation dynamics (25). The PCR-directed methods dominating molecular genetic analysis in paleolimnology are useful for reporting partial taxonomic and functional components of historical microbiomes. However, reconstructing historical diversity through PCR amplicon sequencing is limited by taxonomic blind-spots (26), restricted gene quantification value (27), and dependency on intact DNA template (28). Furthermore, many major microbial groups have never been targeted in standard single-gene surveys, leaving unexplored any new insights they might yield on past lake ecosystems.

Metagenomics has the potential to provide a powerful new perspective to probe the unconstrained diversity preserved in sediment archives. Shotgun sequencing has gained traction in other ancient DNA disciplines (e.g., studies of ancient hominins in evolutionary anthropology) to recover ultrashort DNA fragments not amplifiable by PCR and to avoid other PCR biases (29). To date, only a handful of paleolimnological studies have applied shotgun sequencing. Pedersen et al. reconstructed the postglacial succession of plants, algae, and animals from lake sediment metagenomes (30). Subsequent research demonstrated that shotgun sequencing expands the richness of catchment flora detected in lake sediments (31) and that ancient chloroplast and mitochondrial genomes can be resolved from the DNA remains of vegetation (32) and algae (33). Aside from the studies on eukaryotic algae, the only other paleolimnological reconstruction to describe autochthonous microbiomes with shotgun metagenomics profiled the succession of archaea in an ancient lake (34). In these studies, historical DNA represented tiny fractions of sediment metagenomes but still furnished new information (i.e., not overlapping with classical proxies) about past lacustrine or catchment biota. As these works explore the potential of metagenomics, they underscore the primary challenges associated with reconstructing historical records from sediment metagenomes. Sediment DNA pools derive foremost from indigenous sediment microbes, and historical DNA is subject to high turnover (35). Historical signals are difficult to detect because they are weaker than, and are distorted by, indigenous sediment populations.

In this study, whole-metagenome shotgun sequencing was used to probe the diversity of microorganisms preserved in contemporary and preindustrial sediment archives to expand the taxonomic scope of lake microbiome reconstructions. To identify historical DNA in sediment metagenomes, microbial preservation signals were captured by mapping sediment metagenome reads to surface water metagenome assemblies. This fragment recruitment strategy was used to disentangle historical DNA from the indigenous sediment background, using the contemporary diversity in the overlying water column as a reference. The detection of preserved DNA archives was subsequently expanded in the unconstrained explorations of whole-sediment metagenomes, guided by leads developed through metagenome capture. By teasing out the DNA preservation signals of historical lake microbiomes in the sediment record, we are able to harness a more complete perspective on the diversity of lakes and their ecological functioning over decades to millennia.

## RESULTS

### Environmental context.

**FIG 1:**
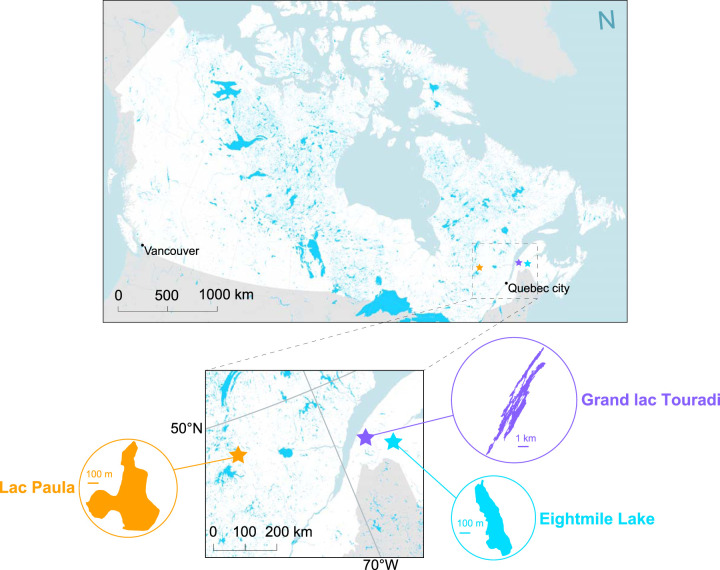
Map of Canada with the starred locations of study lakes: Lac Paula, Eightmile Lake, and Grand lac Touradi. The map was created in QGIS v. 3.10 (77) with the Canada Atlas Lambert projection and shapefile data sourced for the hydrographic features (78) and political boundaries of Canada (79). Spatial coordinates, morphological characteristics, and physicochemical profiles of study lakes are summarized in Table 1.

Whole-metagenome shotgun sequencing was performed on surface water and sediment DNA sampled from three regionally representative eastern Canadian lakes: Lac Paula, Eightmile Lake, and Grand lac Touradi (Fig. 1; Table 1). Lac Paula is a medium-sized, deep lake set in a small, equal parts clear-cut and naturally forested watershed in central Quebec in the Boreal Shield ecozone. Eightmile Lake is a small lake of intermediate depth set in a small, ∼38% clear-cut watershed in northern New Brunswick in the Atlantic Highlands ecozone. Grand lac Touradi is a large, deep lake in an expansive, naturally forested watershed in eastern Quebec in the Atlantic Highlands ecozone. Top sediments were sampled from the recently deposited, upper 1 cm of each core, and bottom sediments were sampled from preindustrial deposits (on average, 40 cm below the water-sediment interface). A conservative lead ^210^Pb (lead-210) dating model placed all bottom sediment ages in the preindustrial background (i.e., pre-1880 Common Era [C.E.]) (see Fig. S1 in the supplemental material).

**TABLE 1:** Spatial coordinates, morphological characteristics, and surface water physicochemical profiles of study lakes

Lake	Latitude (°N)	Longitude (°W)	Maximum depth (m)	Surface area (km^2^)	Watershed area (km^2^)	Chlorophyll a concn (mg/liter)	Dissolved inorganic carbon concn (mg/liter)	Dissolved organic carbon concn (mg/liter)	Soluble reactive phosphorus concn (μg/liter)
Lac Paula	48.991254	74.028099	17.0	0.6	3.2	1.7	1.1	5.9	3
Eightmile Lake	47.694086	67.642841	4.5	0.2	1.7	3.5	14.6	6.5	2
Grand lac Touradi	48.131057	68.666779	17.0	7.7	117.7	2.6	15.6	6.5	3

10.1128/mSphere.00512-20.1 FIG S1 Preindustrial (i.e., pre-1880) bottom sediment depositional ages were inferred when overlap was observed between bottom sediment lead-210 and bismuth-214 activities within 1 standard deviation. Download FIG S1, PDF file, 0.2 MB. Copyright © 2020 Garner et al. 2020 Garner et al. This content is distributed under the terms of the Creative Commons Attribution 4.0 International license.

### Preserved metagenome capture.

**FIG 2:**
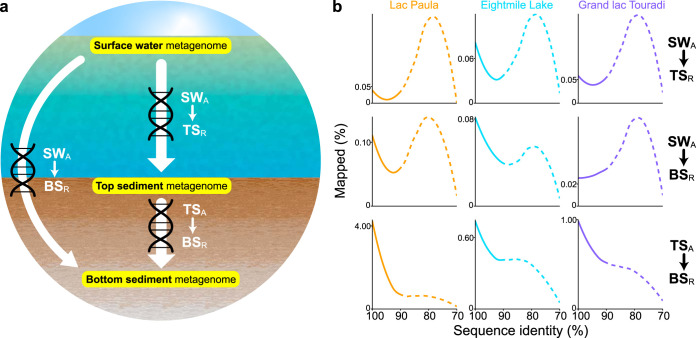
(a) As illustrated in this experimental design schematic, the recruitment of unassembled metagenome reads (abbreviated subscript R) to metagenome assemblies (subscript A) tracks the sequence similarities between focal and reference metagenomes. Captured metagenomes are abbreviated SW_A_ → TS_R_ (top sediment reads mapped to the surface water assembly), SW_A_ → BS_R_ (bottom sediment reads mapped to the surface water assembly), and TS_A_ → BS_R_ (bottom sediment reads mapped to the top sediment assembly). Arrows point in the direction of DNA preservation, from the overlying source to the underlying sediment archive. (b) Metagenome capture was performed with a 90% sequence identity threshold derived from the delineation of closely (solid curve, ≥90%) and distantly (dashed curve, <90%) related sequences. Reads mapped to scaffolds containing ribosomal or transfer RNA genes are included (exceptionally) in this figure, with negligible impact on the results presented (see Table S1 in the supplemental material).

We define captured metagenomes as products of *in silico* read recruitment and free metagenomes as whole metagenomes unconstrained by capture. Microbial DNA preserved in sediment metagenomes was captured by mapping top and bottom sediment metagenome reads to surface water metagenome assemblies (Fig. 2a). Some low-similarity alignment was expected to arise from distant evolutionary relationships between freshwater microorganisms and indigenous sediment populations. To disentangle historical and indigenous sediment microbiome DNA, capture of closely related sequences was performed with a stringent 90% identity threshold, empirically derived from the consistent delineation of closely (≥90%) and distantly (<90%) related populations (Fig. 2b). Most scaffolds in captured metagenomes did not contain highly conserved ribosomal or transfer RNA genes (97.9% ± 1.0%) (see Table S1 in the supplemental material). Still, to avoid overinflating the estimates of DNA preservation from false-positive detections of distantly related organisms aligned at conserved sequences, scaffolds containing ribosomal or transfer RNA genes were removed (Table S1). Mapping between sediment and surface water metagenomes was detected across all lakes (0.2 to 0.8%), but it was lower than mapping between bottom and top sediment metagenomes (5.5 to 21.0%) (Table 2). On average, 98.9% ± 0.8% of scaffolds in captured metagenomes contained at least one annotated gene, and the majority of scaffolds across captured metagenomes contained a single gene (56.9% ± 11.8%) (Fig. S2a).

**TABLE 2:** Counts and percentages (normalized by the number of unassembled reads) of reads mapped with a 90% sequence identity threshold in metagenome capture experiments

Lake	Captured metagenome^*a*^	Unassembled read count	Mapped read count	Mapped (%)
Lac Paula	SW_A_ → TS_R_	32,622,245	69,318	0.2
	SW_A_ → BS_R_	67,840,581	511,831	0.8
	TS_A_ → BS_R_	67,840,581	14,234,026	21.0
Eightmile Lake	SW_A_ → TS_R_	29,873,977	145,692	0.5
	SW_A_ → BS_R_	29,740,441	188,125	0.6
	TS_A_ → BS_R_	29,740,441	1,622,267	5.5
Grand lac Touradi	SW_A_ → TS_R_	29,290,940	143,854	0.5
	SW_A_ → BS_R_	30,470,979	97,815	0.3
	TS_A_ → BS_R_	30,470,979	2,324,829	7.6

a Captured metagenome abbreviations are defined in the legend to Fig. 2a.

10.1128/mSphere.00512-20.2 FIG S2 (a) Frequencies of scaffolds containing *n* genes. (b) Average fold and lengths of scaffolds in captured metagenomes. Download FIG S2, PDF file, 1.6 MB. Copyright © 2020 Garner et al. 2020 Garner et al. This content is distributed under the terms of the Creative Commons Attribution 4.0 International license.

10.1128/mSphere.00512-20.6 TABLE S1 Comparisons of the number of scaffolds and mapped reads in captured metagenomes before and after scaffolds containing ribosomal or transfer RNA genes were removed. Download Table S1, PDF file, 0.02 MB. Copyright © 2020 Garner et al. 2020 Garner et al. This content is distributed under the terms of the Creative Commons Attribution 4.0 International license.

### Taxonomic diversity in free and captured metagenomes.

**FIG 3:**
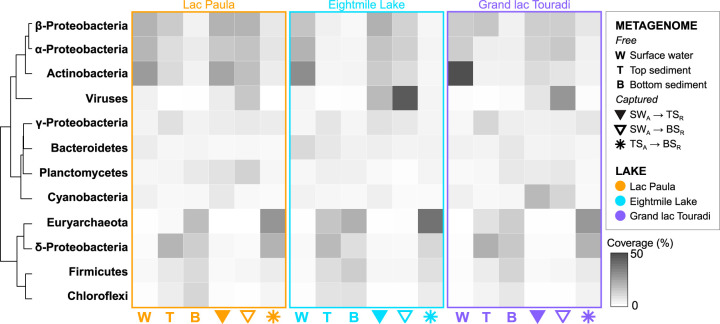
Relative coverage of prevalent phyla in the free and captured metagenomes of three lakes. Represented in this heat map are taxa with ≥10% relative coverage in at least one metagenome.

We performed taxonomic assessments of free and captured metagenomes to compare the compositions of natural surface water and sediment microbiomes with the microbial diversity preserved in sediment DNA archives (Fig. 3; Table S2). Free surface water metagenomes in all lakes contained high relative coverage of *Actinobacteria*, *Betaproteobacteria*, *Alphaproteobacteria*, and *Bacteroidetes*. Free top sediment metagenomes also contained high relative coverage of *Actinobacteria* and *Betaproteobacteria* but were also rich in Deltaproteobacteria, *Euryarchaeota*, and *Gammaproteobacteria*. Free bottom sediment metagenomes contained high relative coverage of *Euryarchaeota*, Deltaproteobacteria, *Firmicutes*, and *Chloroflexi*.

10.1128/mSphere.00512-20.7 TABLE S2 Relative coverage of phyla in the free and captured metagenomes of three lakes. Download Table S2, PDF file, 0.1 MB. Copyright © 2020 Garner et al. 2020 Garner et al. This content is distributed under the terms of the Creative Commons Attribution 4.0 International license.

Analyses of the captured metagenomes considered at the phylum level demonstrated a relatively consistent assemblage of preserved microbiota, with a few differences between contemporary and preindustrial sediments (Fig. 3; Table S2). Comparisons of the captured surface water signals in either top or bottom sediment metagenomes contained high relative coverage of viruses, *Actinobacteria*, *Betaproteobacteria*, *Cyanobacteria*, and *Alphaproteobacteria*. Captured surface water signals in bottom sediment metagenomes also contained high relative coverage of *Planctomycetes*. Captured top sediment signals in bottom sediment metagenomes contained high relative coverage of *Euryarchaeota* and Deltaproteobacteria.

**FIG 4:**
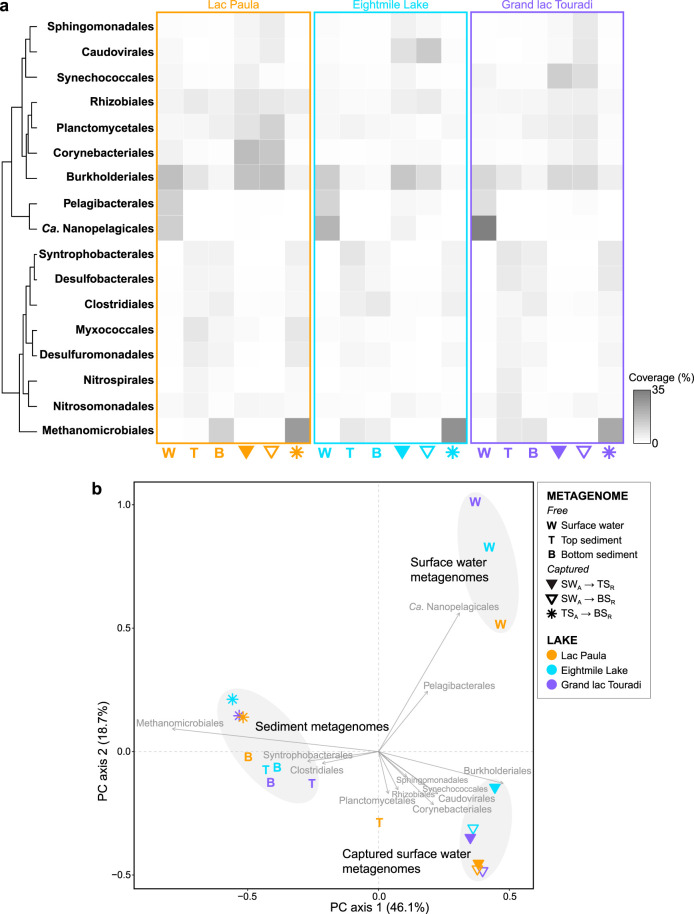
(a) Relative coverage of prevalent taxonomic orders in the free and captured metagenomes of three lakes. Represented in this heat map are taxa with ≥5% relative coverage in at least one metagenome. (b) PCA of order-rank taxonomic composition data of free and captured metagenomes. The predominant taxon contributions to the first two PC axes are shown. Groups of taxonomically similar metagenomes are highlighted by grey-shaded ellipses.

To elucidate the taxonomic diversity of free and captured metagenomes at higher phylogenetic resolution, taxonomic analyses were performed at the order level (Fig. 4a; Table S3). A principal-component analysis (PCA) of these data demonstrated a clear distinction between free and captured metagenomes along the first principal component (PC) axis, reflecting the taxonomic distinctness of surface water and sediment microbiomes (Fig. 4b). Free and captured surface water metagenomes were separated along the second PC axis, suggesting that only a subset of microbial diversity is preserved in sediments (Fig. 4b). The first two PC dimensions together explained 64.8% of the variation between metagenome taxonomic compositions. “*Candidatus* Nanopelagicales” and *Pelagibacterales* were highly covered in the free surface water metagenomes of all lakes, but they were not highly covered in captured surface water metagenomes (Fig. 4a; Table S3). Free and captured sediment metagenomes contained high relative coverage of *Methanomicrobiales*, *Syntrophobacterales*, *Desulfobacterales*, *Clostridiales*, *Desulfuromonadales*, *Myxococcales*, *Nitrosomonadales*, *Rhizobiales*, and *Nitrospirales* (Fig. 4a; Table S3).

10.1128/mSphere.00512-20.8 TABLE S3 Relative coverage of prevalent taxonomic orders (≥5% relative coverage in at least one metagenome) in the free and captured metagenomes of three lakes. Download Table S3, PDF file, 0.05 MB. Copyright © 2020 Garner et al. 2020 Garner et al. This content is distributed under the terms of the Creative Commons Attribution 4.0 International license.

### Highlights of preservation signals in captured metagenomes.

Since most freshwater *Cyanobacteria* are photosynthetic, *Cyanobacteria* DNA detected in the metagenomes of subsurface sediments can be distinguished from the indigenous sediment background and identified as the preserved DNA of *Cyanobacteria* that inhabited historical sunlit lake biomes. Captured surface water metagenomes exhibited higher relative coverage of *Cyanobacteria* than free surface water metagenomes, highlighting recruitment capture as a viable strategy for detecting well-preserved historical microorganisms (Fig. 3; Table S2). Of the three lakes, Grand lac Touradi displayed the most sizable relative capture of preserved *Cyanobacteria* in contemporary and preindustrial sediments (Fig. 3; Table S2). *Synechococcales* were the most highly represented *Cyanobacteria* in captured surface water metagenomes (Fig. 4a; Table S3).

Captured surface water metagenomes also showed that sediments accumulate DNA from some—but not all—bacterioplankton, as well as viruses. The most highly covered bacterioplankton that were represented in captured surface water metagenomes were *Corynebacteriales*, *Burkholderiales*, *Planctomycetales*, *Sphingomonadales*, and *Rhizobiales* (Fig. 4a; Table S3). *Burkholderiales* were highly covered in both free and captured surface water metagenomes and in free top sediment metagenomes, suggesting that some planktonic *Burkholderiales* are preserved in sediments, while other *Burkholderiales* are indigenous to sediments and may potentially confound historical signals without higher-resolution taxonomic assessments (Fig. 4a; Table S3). Some highly abundant bacterioplankton (*Ca*. Nanopelagicales, *Pelagibacteriales*) were only marginally covered in captured surface water metagenomes (Fig. 4a; Table S3). Viruses were highly covered in the captured surface water metagenomes of all lakes, but they were not highly covered in the free surface water metagenomes (Fig. 3; Table S2). *Caudovirales* were the most highly captured viruses, especially in Eightmile Lake (Fig. 4a; Table S3). A phylogeny inferred from the terminase large subunit protein (TerL) identified a wide diversity of *Caudovirales*, but host identities could not be resolved (Fig. S3).

10.1128/mSphere.00512-20.3 FIG S3 Terminase large subunit protein (TerL) phylogeny of *Caudovirales* detected in free and captured metagenomes. Download FIG S3, PDF file, 0.3 MB. Copyright © 2020 Garner et al. 2020 Garner et al. This content is distributed under the terms of the Creative Commons Attribution 4.0 International license.

### Exploring unconstrained preservation signals in free metagenomes.

**FIG 5:**
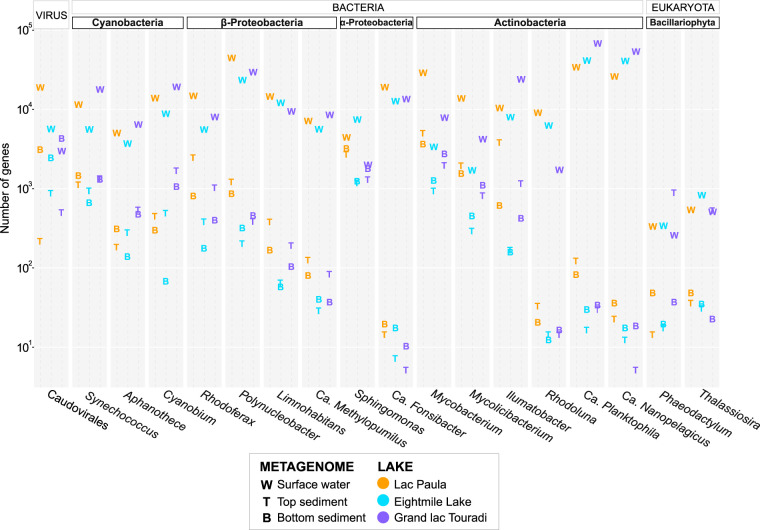
Gene counts of selected preserved *Cyanobacteria*, bacterioplankton, microeukaryotes, and viruses in free surface water and sediment metagenomes in three lakes.

While metagenome capture was practical for developing leads on preserved taxa, experimental limitations rendered this approach unsuitable for recruiting unconstrained preservation signals for historical lake microbiomes. Specifically, the seasonal, depth-discrete, and contemporary community structures of surface water snapshot samples constrained the capture of a broad microbial diversity. To expand the detection of preserved DNA to include the diversity that escaped capture, the search for preserved taxa was extended to the unconstrained DNA libraries of free sediment metagenomes. Across all lakes, free top and bottom sediment metagenomes contained traceable accumulations of genes from *Cyanobacteria*, bacterioplankton, and microeukaryotes (Fig. 5). Genes assigned to *Cyanobacteria* (*Synechococcus*, *Aphanothece*, and *Cyanobium*) numbered in the thousands in free surface water metagenomes and were detected to a lesser extent in top and bottom sediment metagenomes (Fig. 5). Sediment metagenomes contained genes from other bacterioplankton, including *Betaproteobacteria* (*Rhodoferax*, *Polynucleobacter*, and *Limnohabitans*), *Alphaproteobacteria* (*Sphingomonas*), *Actinobacteria* (*Mycobacterium*, *Mycolicibacterium*, *Ilumatobacter*, and *Rhodoluna*) (Fig. 5), and others (Fig. S4). A small number of genes from eukaryotic plankton, namely, diatoms (*Phaeodactylum* and *Thalassiosira*), were also detected in sediment metagenomes (Fig. 5). Some bacterioplankton (“*Candidatus* Methylopumilus,” “*Candidatus* Fonsibacter,” “*Candidatus* Planktophila,” and “*Candidatus* Nanopelagicus”) that were highly covered in surface water metagenomes were represented by relatively low gene counts in free sediment metagenomes (Fig. 5).

10.1128/mSphere.00512-20.4 FIG S4 Extension of Fig. 5 to include all genus-rank taxa with ≥1% coverage in free or captured surface water metagenomes. Download FIG S4, PDF file, 0.4 MB. Copyright © 2020 Garner et al. 2020 Garner et al. This content is distributed under the terms of the Creative Commons Attribution 4.0 International license.

To demonstrate how preserved microbial diversity might be identified at high phylogenomic resolution, surface water and sediment metagenome reads were mapped to publicly available reference genomes from *Polynucleobacter* and *Limnohabitans*, two genera of *Betaproteobacteria* with well-described freshwater microdiversity and that were common in captured metagenomes. Surface water metagenomes from Eightmile Lake and Grand lac Touradi contained *Polynucleobacter* and *Limnohabitans* populations with high sequence similarity (≥90%) to reference genomes (Fig. S5). In contrast, reference genomes were not captured in any of the top or bottom sediment metagenomes (Fig. S5).

10.1128/mSphere.00512-20.5 FIG S5 Recruitment of surface water and sediment metagenome reads to concatenated *Polynucleobacter* and *Limnohabitans* reference genomes. Download FIG S5, PDF file, 0.3 MB. Copyright © 2020 Garner et al. 2020 Garner et al. This content is distributed under the terms of the Creative Commons Attribution 4.0 International license.

## DISCUSSION

This study illustrates that shotgun sequencing can access a broad diversity of microorganisms preserved in contemporary and preindustrial lake sediments. Preserved taxa were identified by mapping sediment metagenome reads to reference surface water metagenome assemblies with a stringent 90% sequence identity threshold derived to disentangle freshwater signals from the indigenous sediment background. Once well-preserved lineages were identified through the *in silico* capture of surface water signals in sediment metagenomes, unconstrained explorations for preserved taxa were conducted in free sediment metagenomes to uncover preserved diversity that escaped capture. This strategy recovered a wide diversity of freshwater microorganisms previously overlooked by more classical paleolimnological approaches. Given that microorganisms have critical functions in lake ecosystems, including carbon and other elemental cycling, studying the dynamics of lake microbiomes in the past could be quite insightful.

### Preserved taxa in sediment metagenomes.

Taxonomic analysis of contemporary and preindustrial sediment metagenomes showed that these metagenomes contained hundreds to thousands of preserved genes from freshwater microbiota within the *Cyanobacteria* (*Synechococcus*, *Aphanothece*, and *Cyanobium*), *Betaproteobacteria* (*Rhodoferax*, *Polynucleobacter*, and *Limnohabitans*), *Alphaproteobacteria* (*Sphingomonas*), *Actinobacteria* (*Mycobacterium*, *Mycolicibacterium*, *Ilumatobacter*, and *Rhodoluna*), microeukaryotes (*Phaeodactylum* and *Thalassiosira*), and viruses (*Caudovirales*).

*Betaproteobacteria* were abundant in surface waters and well preserved in top and bottom sediment metagenomes. The ubiquity of *Burkholderiales* in water column and sediment microbiomes illustrates the importance of applying a stringent sequence identity cutoff to capture the elements of sediment metagenomes closely related to freshwater taxa as an approach to identifying historical DNA. *Burkholderiales* exhibit wide physiological and ecological diversity and have been isolated from a range of different sources, including soils and freshwater lakes (36, 37). The sheer diversity of *Burkholderiales* makes it difficult to pin down specific traits which can explain their preservation in sediment metagenomes.

In general, taxa were not equally preserved in sediment metagenomes. *Actinobacteria* and *Alphaproteobacteria* were numerically important in surface water metagenomes, reflecting distribution patterns typical of freshwater lakes (38, 39). Despite dominating surface water microbiomes, *Ca*. Nanopelagicales (*Ca.* Nanopelagicus and *Ca.* Planktophila), *Pelagibacterales* (*Ca.* Fonsibacter), and other ubiquitous bacterioplankton (*Ca.* Methylopumilus) were not well preserved in top or bottom sediments. *Ca*. Nanopelagicales, *Pelagibacterales*, and *Ca.* Methylopumilus are ultramicrobacteria characterized by free-living lifestyles and streamlined genomes with low guanine-cytosine (GC) contents (40–42), traits held in common with the well-preserved ultramicroscopic *Burkholderiales*. A variety of mechanisms has been put forth to explain the differential DNA preservation across microbial groups previously observed in single-gene time series, including sporulation (43), cellular architecture and membrane biochemistry (44), genome GC content (45), and autoecology. However, further work is needed to elucidate how DNA is differentially preserved across taxa and under different lake conditions.

The robust preservation of viruses in the captured metagenomes of all three lakes suggests that historical viromes are at least partially accessible to reconstruction from sediment archives. The best preserved viruses were *Caudovirales*, tailed bacteriophages infecting a wide range of hosts through both lytic and lysogenic replication (46). A phylogeny based on TerL appeared to resolve the *Caudovirales* as phages associated with several bacterial taxa; however, the tree lacked both the strong bootstrap confidence and clear delineation of clades necessary for definitive host identifications. The strong preservation of bacteriophage DNA is likely related to two mechanisms. First, prophages are widely integrated in the genomes of infected microorganisms (47), whose own DNA is variably preserved in sediments. Paleoecological time series can exploit the concurrent preservation of viral and host DNA to reconstruct virus-host evolution and illuminate the influence of environmental change on virus-host dynamics (48). The second mechanism of preservation is the hardiness of encapsidated virions, which are suited to withstand adverse conditions and remain intact under long-term cold storage (49). As abundant biological entities that exert enormous control over microbial populations in aquatic ecosystems (50, 51), viruses could lend valuable insights into historical lake microbiomes.

A major issue in ancient DNA disciplines is that historical signals can be distorted or diluted by cross-contamination from contemporary DNA (14). The exposure of sediment DNA to modern contaminants can potentially occur at any stage of sediment core sampling or laboratory work. The first precaution we took to minimize cross-contamination was to retrieve the innermost sediments furthest from the core tube walls at top and bottom intervals using sterile spatulas and syringes. Another precaution was to perform DNA extractions from water filtrates and sediments in separate dedicated laboratories under positive-pressure UV hoods. Precautions aside, the historical microbial signals detected in sediment metagenomes appeared valid and not contaminated by contemporary DNA, as evidenced by the observed trends in DNA preservation across groups. If sediment metagenomes were contaminated by surface water DNA, we would expect the preferential recruitment of sediment metagenome reads to DNA from common and abundant lacustrine microorganisms (e.g., ultramicroscopic *Actinobacteria* and *Alphaproteobacteria*). Instead, preferential recruitment of sediment metagenome reads to rarer surface water taxa was observed.

### Harnessing microbial ecotypes as paleoindicators.

The detection of preserved DNA from a broad diversity of microorganisms expands the taxonomic scope of paleolimnology. To access the paleoindicator potential of preserved microbiota, we suggest that preimpact lake conditions can be reconstructed from records of past microbial ecologies. In the future, paleolimnological reconstructions focused on well-preserved heterotrophic bacteria could provide key insights into past ecosystem functions. The ecologies of ubiquitous lacustrine bacterioplankton are extensively detailed in published literature (38, 52). For some freshwater bacteria, ecotypic diversity has been described, i.e., genetically differentiated lineages with specific ecological requirements (53, 54). We propose that the ecologies of well-studied bacterioplankton, particularly those with resolved ecotypes, can clarify the niche dimensions of historical microbiomes.

Our explorations of captured and free metagenomes detected key bacterioplankton groups for which ecotypic diversity is well described, e.g., *Polynucleobacter* and *Limnohabitans*. The microdiversity of ubiquitous *Polynucleobacter* and *Limnohabitans* has been investigated in a wide variety of freshwater lakes and has been physiologically characterized through culture, transplant experiments, and genome sequencing (55–58). The niche specificities of *Polynucleobacter* or *Limnohabitans* ecotypes in DNA time series could potentially describe historical water column conditions (e.g., pH, carbon and other nutrient substrates, light penetration, temperature, and oxygen levels). Historical ecotypes might be identified by mapping sediment metagenomes to ecotype reference genomes, or a reduced approach might focus on recovering marker genes with sufficient phylogenetic information to resolve microdiversity. Toward the identification of ecotypes, we attempted to map sediment metagenomes to publicly available *Polynucleobacter* and *Limnohabitans* reference genomes. While reference genomes were captured in the surface water metagenomes of Eightmile Lake and Grand lac Touradi, they were not captured in the sediment metagenomes. The low-similarity alignment of sediment metagenomes and reference genomes representing genera detected in sediment metagenomes suggests that these contemporary reference genomes do not reflect historical microdiversity. Ecotypes could potentially be resolved if sets of genomes or metagenome-assembled genomes representative of local to regional pre- and postimpact diversity were made available, especially through large-scale metagenomic lake surveys (59). Comparing the ecological dependencies reconstructed in ecotype time series could then retrace environmental change.

### Preserved metagenome capture.

Metagenome capture was designed to frame a novel methodology and proof of concept for the detection of historical DNA with shotgun metagenomics. However, with the approach we adopted in this pilot study, we are limited in our abilities to represent the totality of preserved microbial diversity. First, our surface water metagenomes were snapshot samples of DNA collected from the photic zone during summer stratification. These reference metagenomes comprised microbial assemblages partially representative of the vertical structure of stratified lakes, which varies seasonally. Second, surface water metagenomes represented contemporary diversity. Some degree of community turnover is expected across the more than 150 years separating preindustrial and contemporary microbiomes, and preindustrial diversity no longer occurring in contemporary microbiomes cannot be recruited. Finally, metagenome assembly only partly incorporated unassembled metagenomes, with the effect that reference assemblies further constricted the scope of sampled diversity. These limitations parametrizing the capture of snapshot surface water metagenomes effectively set up the capture of preservation signals to fail. Yet even so, we recovered partial preserved metagenomes from sediments with novel taxonomic information.

Metagenome capture was most prolific between sediment metagenomes, which displayed 20- to 30-fold-higher sequence mapping than captured surface water metagenomes. The continuity between top and bottom sediment metagenomes was observed in each lake, despite a depositional age difference of over 150 years and commonly observed steep geochemical gradients along sediment horizons (60). In contrast, the high taxonomic variation between surface water and sediment metagenomes can be explained by the stark environmental dissimilarities between photic zone and sediment subsurface habitats (61) and by the low composition of sediment DNA that is of historical origin (35). Experimental capture showed that very small fractions of sediment metagenomes originated from historical freshwater microorganisms (<1%, under the limitations of recruitment to snapshot reference surface water metagenomes). The disparity between the proportions of indigenous and historical DNA in sediments underscores the need to develop strategies to optimize historical DNA recovery while cancelling out signals from background sediment populations. One obvious path forward would be to apply the capture approach on a combined assembly of reference water column metagenomes, collected over a larger set of lakes or across multiple seasons. Nonetheless, the historical DNA detected by metagenome capture was sufficient to partially reconstruct historical lake microbiomes. PCR gene amplification is likely the most practical approach for targeting the population dynamics of preserved groups identified in this study. Future works might also attempt to extract historical signals embedded in indigenous sediment microbiomes (62, 63).

### Conclusion and outlook.

In conclusion, we put forth two ideas to meet some of the unrealized potential of molecular genetic analysis in paleolimnology. First, whole-metagenome shotgun sequencing can access a broad diversity of microorganisms preserved in lake sediment DNA archives. Second, microorganisms are underexploited paleoindicators whose ecotypic diversity can potentially retrace environmental flux. This study tested a proof of concept that DNA from historical lake microbiomes can be recovered from sediment metagenomes. Our strategy of baiting unassembled sediment metagenome reads with reference surface water metagenome assemblies was designed to disentangle historical microbial signals from the indigenous sediment background. Future works are encouraged to improve on this approach to optimize the recovery of historical signals, e.g., by querying population-level gene variation in microbial time series with PCR amplification. All told, our application of shotgun DNA sequencing has expanded the detectable microbial diversity available to paleolimnological reconstructions to include heterotrophic bacteria and viruses, which we posit are as important in historical contexts as they are in contemporary lake microbiomes.

## MATERIALS AND METHODS

### Lake water and sediment sampling.

Surface water and sediment cores were sampled from three eastern Canadian lakes between 31 July and 25 August 2017 during the Canadian Lake Pulse Network field campaign (59) (Fig. 1; Table 1). Lakes were selected by meeting the criteria of a maximum depth of at least 1 m and accessibility within 1 km from a road (59). At each lake, surface water and sediment cores were collected at the site of maximum depth, located by depth sounding and using bathymetric maps as guides when available. All water sampling devices were acid washed and triple rinsed before sample processing. Water was collected from the euphotic zone over an integrated depth of up to 2 m below the surface with an integrated tube sampler. Carboys were stored in chilled coolers until same-day water filtration on shore. Up to 500 ml of water was prefiltered through 100-μm synthetic nylon (Nitex) mesh and then vacuum filtered on 47-mm-diameter 0.22-μm Durapore membranes (MilliporeSigma, Darmstadt, Germany) through a glass funnel apparatus at a maximum pressure of 8 in Hg. Sediment core target length was estimated to reach preindustrial depositional age based on regional sedimentation rates synthesized from the literature. Sediment cores were retrieved using a gravity corer fitted with a 68-mm-diameter plastic core tube. Sediment cores were extruded on shore and subsampled at top and bottom 1-cm intervals. Bottom sediment subsections collected at core depth below the water-sediment interface were relatively consistent: the 40- to 41-cm interval in Lac Paula and the 39- to 40-cm interval in Grand lac Touradi and Eightmile Lake. Samples for molecular genetic analysis were collected from innermost sediments, furthest from the walls of the core tube, using a sterile syringe or spoon. Water filters and sediment samples were stored at −80 and −20°C, respectively. To determine the depositional ages of bottom sediments, 210Pb and bismuth-214 (214Bi) radionuclide activities were measured using gamma spectrometry and a constant rate of supply model (64). When overlap was observed between 210Pb and 214Bi activities within 1 standard deviation, sediments were determined to have been deposited within the last 100 to 150 years (i.e., reaching preindustrial background).

### DNA extraction.

DNA was extracted from filters using the DNeasy PowerWater kit (Qiagen, Hilden, Germany) according to the manufacturer’s instructions with the addition of the following optional steps: after vortex bead beating and centrifugation (step 7 of manufacturer’s detailed protocol), 1 μl of RNase A was added to samples before a 30-min incubation at 37°C. DNA extractions from sediments were performed in a UV cabinet in a separate dedicated laboratory. DNA was extracted from 0.500 ± 0.015 g of wet sediment using the NucleoSpin soil kit (Macherey-Nagel, Düren, Germany) according to the manufacturer’s instructions with lysis buffer SL1 and enhancer SX options, without protocols for separating intra- and extracellular DNA. DNA was quantified using the Qubit dsDNA BR assay kit (Invitrogen, Carlsbad, CA, USA) in a Qubit 2.0 fluorometer.

### Metagenome shotgun sequencing, assembly, and annotation.

DNA samples were submitted to Genome Quebec for library barcoding and shotgun sequencing of 150-bp paired-end reads on an Illumina NovaSeq 6000 platform with flow cell type S2. The sequencing depth ranged from 25,717,497 to 73,554,802 reads per sample. Sequencing adapters, low-quality bases, and ultrashort reads were trimmed with Trimmomatic v. 0.38 (65). Metagenome assembly from paired reads was performed with MEGAHIT v. 1.0.6 using k-mer lengths 23, 43, 63, 83, 103, and 123 (66). Assemblies were submitted to the Integrated Microbial Genomes & Microbiomes (IMG) system for annotation in IMG Annotation Pipeline v. 4.16.6 (67). Taxonomy was predicted by phylodist (i.e., the taxonomic affiliation of the closest gene homolog) in the IMG pipeline.

### Read recruitment.

For each lake, read recruitment was conducted between surface water, top sediment, and bottom sediment metagenomes, always in the direction tracking the downward preservation of DNA (i.e., reads from the underlying metagenome were mapped to the overlying metagenome assembly; Fig. 2a). Global read recruitment was performed with BBMap v. 35.68 (68) using an initial, highly permissive nucleotide sequence identity threshold of ∼70% to assess alignment trends between metagenomes. A subsequent, empirically derived sequence identity threshold of 90% was applied to align closely related sequences. This cutoff was selected after observing consistent shifts in recruitment trend across capture experiments toward 90% sequence identity, which appeared to delineate closely (≥90%) and distantly (<90%) related populations (Fig. 2b). To reject false-positive results overestimating DNA preservation, scaffolds containing ribosomal or transfer RNA genes were removed from captured metagenomes because these conserved sequences are shared with relatively high similarity among distantly related organisms and can produce biased alignment. The number of mapped reads was normalized by the number of unassembled reads in the recruitment effort. Scaffold lengths and gene counts on scaffolds in captured metagenomes were evaluated to determine whether the taxonomic annotations of protein-coding sequences could be extended to taxonomic assignments of whole scaffolds (see Fig. S2 in the supplemental material).

To increase the taxonomic resolution for groups with well-described freshwater microdiversity, surface water and sediment metagenome reads were recruited to *Polynucleobacter* and *Limnohabitans* reference genomes available from IMG (listed in Table S4 in the supplemental material). Ribosomal and tRNA genes were masked in reference genomes with BEDTools maskfasta v. 2.26.0 (69) to avoid read recruitment from distantly related organisms to conserved sequences. Read recruitment from surface water and sediment metagenomes to concatenated reference genomes was performed in BBMap with the default ∼70% sequence identity threshold in order to visualize recruitment success as a function of genetic similarity.

10.1128/mSphere.00512-20.9 TABLE S4 Accession information for *Betaproteobacteria* reference genomes downloaded from IMG. Download Table S4, PDF file, 0.1 MB. Copyright © 2020 Garner et al. 2020 Garner et al. This content is distributed under the terms of the Creative Commons Attribution 4.0 International license.

### Statistical analyses.

Principal-component analysis (PCA) was performed on metagenome taxonomic composition data to evaluate variation among captured and free metagenomes from all study lakes. Relative coverage-weighted metagenome composition data of order-rank taxonomy were chord-transformed with the function decostand(method = “norm”) in the R package vegan (70). The PCA was computed with the vegan function rda(). Dendrograms of prevalent taxa were based on Ward’s hierarchical clustering of the Bray-Curtis dissimilarities between metagenome taxonomic compositions across lakes. Data wrangling and visualization were programmed in R (71) using the tidyverse package suite (72).

### Phylogenetic analyses.

To resolve the host identities of captured bacteriophages, a *Caudovirales* phylogeny was constructed based on TerL, a common taxonomic marker for this group (73). *Caudovirales* TerL amino acid sequences detected in metagenomes were aligned with publicly available reference sequences in MAFFT online service using default settings (74). A maximum likelihood tree was inferred using the Jones-Taylor-Thornton substitution model (75) with 5 discrete gamma categories and 100 bootstrap replicates in MEGA-X (76).

### Data availability.

The annotated metagenome assemblies in this study are available from the Integrated Microbial Genomes & Microbiomes system (U.S. Department of Energy Joint Genome Institute, Berkeley, CA, USA) at https://img.jgi.doe.gov under GOLD Study Gs0136026. Links to the GOLD Analysis Projects are listed in Table S5. All scripts are available at https://github.com/rebeccagarner/paleocapture.

10.1128/mSphere.00512-20.10 TABLE S5 GOLD Analysis Project identifiers (IDs) and links for annotated metagenome assemblies in this study. Download Table S5, PDF file, 0.02 MB. Copyright © 2020 Garner et al. 2020 Garner et al. This content is distributed under the terms of the Creative Commons Attribution 4.0 International license.

## References

[1] Tranvik LJ, Downing JA, Cotner JB, Loiselle SA, Striegl RG, Ballatore TJ, Dillon P, Finlay K, Fortino K, Knoll LB, Kortelainen PL, Kutser T, Larsen S, Laurion I, Leech DM, Leigh McCallister S, McKnight DM, Melack JM, Overholt E, Porter JA, Prairie Y, Renwick WH, Roland F, Sherman BS, Schindler DW, Sobek S, Tremblay A, Vanni MJ, Verschoor AM, Von Wachenfeldt E, Weyhenmeyer GA 2009 Lakes and reservoirs as regulators of carbon cycling and climate. Limnol Oceanogr 54:2298–2314. doi:10.4319/lo.2009.54.6_part_2.2298.

[2] Anderson NJ, Heathcote AJ, Engstrom DR, Globocarb data contributors. 2020 Anthropogenic alteration of nutrient supply increases the global freshwater carbon sink. Sci Adv 6:eaaw2145. doi:10.1126/sciadv.aaw2145. 32494589 PMC7159926

[3] Williamson CE, Dodds W, Kratz TK, Palmer MA 2008 Lakes and streams as sentinels of environmental change in terrestrial and atmospheric processes. Front Ecol Environ 6:247–254. doi:10.1890/070140.

[4] Adrian R, O’Reilly CM, Zagarese H, Baines SB, Hessen DO, Keller W, Livingstone DM, Sommaruga R, Straile D, Van Donk E, Weyhenmeyer GA, Winder M 2009 Lakes as sentinels of climate change. Limnol Oceanogr 54:2283–2297. doi:10.4319/lo.2009.54.6_part_2.2283. 20396409 PMC2854826

[5] Reid AJ, Carlson AK, Creed IF, Eliason EJ, Gell PA, Johnson PTJ, Kidd KA, MacCormack TJ, Olden JD, Ormerod SJ, Smol JP, Taylor WW, Tockner K, Vermaire JC, Dudgeon D, Cooke SJ 2019 Emerging threats and persistent conservation challenges for freshwater biodiversity. Biol Rev Camb Philos Soc 94:849–873. doi:10.1111/brv.12480. 30467930

[6] Dubois N, Saulnier-Talbot É, Mills K, Gell P, Battarbee R, Bennion H, Chawchai S, Dong X, Francus P, Flower R, Gomes DF, Gregory-Eaves I, Humane S, Kattel G, Jenny J, Langdon P, Massaferro J, McGowan S, Mikomägi A, Ngoc NTM, Ratnayake AS, Reid M, Rose N, Saros J, Schillereff D, Tolotti M, Valero-Garcés B 2018 First human impacts and responses of aquatic systems: a review of palaeolimnological records from around the world. Anthrop Rev 5:28–68. doi:10.1177/2053019617740365.

[7] Perga M-E, Frossard V, Jenny J-P, Alric B, Arnaud F, Berthon V, Black JL, Domaizon I, Giguet-Covex C, Kirkham A, Magny M, Manca M, Marchetto A, Millet L, Paillès C, Pignol C, Poulenard J, Reyss J-L, Rimet F, Sabatier P, Savichtcheva O, Sylvestre F, Verneaux V 2015 High-resolution paleolimnology opens new management perspectives for lakes adaptation to climate warming. Front Ecol Evol 3:72.

[8] Giosan L, Orsi WD, Coolen M, Wuchter C, Dunlea AG, Thirumalai K, Munoz SE, Clift PD, Donnelly JP, Galy V, Fuller DQ 2018 Neoglacial climate anomalies and the Harappan metamorphosis. Clim Past 14:1669–1686. doi:10.5194/cp-14-1669-2018.

[9] Smol JP 2019 Under the radar: long-term perspectives on ecological changes in lakes. Proc Biol Sci 286:20190834. doi:10.1098/rspb.2019.0834. 31288704 PMC6650715

[10] Dixit SS, Smol JP, Kingston JC, Charles DF 1992 Diatoms: powerful indicators of environmental change. Environ Sci Technol 26:22–33. doi:10.1021/es00025a002.

[11] Smol JP 1988 Chrysophycean microfossils in paleolimnological studies. Palaeogeogr Palaeoclimatol Palaeoecol 62:287–297. doi:10.1016/0031-0182(88)90058-2.

[12] Handelsman J 2004 Metagenomics: application of genomics to uncultured microorganisms. Microbiol Mol Biol Rev 68:669–685. doi:10.1128/MMBR.68.4.669-685.2004. 15590779 PMC539003

[13] Grossart H, Massana R, McMahon KD, Walsh DA 2020 Linking metagenomics to aquatic microbial ecology and biogeochemical cycles. Limnol Oceanogr 65:S2–S20. doi:10.1002/lno.11382.

[14] Domaizon I, Winegardner A, Capo E, Gauthier J, Gregory-Eaves I 2017 DNA-based methods in paleolimnology: new opportunities for investigating long-term dynamics of lacustrine biodiversity. J Paleolimnol 58:1–21. doi:10.1007/s10933-017-9958-y.

[15] Bálint M, Pfenninger M, Grossart H-P, Taberlet P, Vellend M, Leibold MA, Englund G, Bowler D 2018 Environmental DNA time series in ecology. Trends Ecol Evol 33:945–957. doi:10.1016/j.tree.2018.09.003. 30314916

[16] Falkowski PG, Fenchel T, Delong EF 2008 The microbial engines that drive Earth’s biogeochemical cycles. Science 320:1034–1039. doi:10.1126/science.1153213. 18497287

[17] Glasl B, Webster NS, Bourne DG 2017 Microbial indicators as a diagnostic tool for assessing water quality and climate stress in coral reef ecosystems. Mar Biol 164:91. doi:10.1007/s00227-017-3097-x.

[18] Martínez de la Escalera G, Antoniades D, Bonilla S, Piccini C 2014 Application of ancient DNA to the reconstruction of past microbial assemblages and for the detection of toxic cyanobacteria in subtropical freshwater ecosystems. Mol Ecol 23:5791–5802. doi:10.1111/mec.12979. 25346253

[19] Monchamp ME, Walser JC, Pomati F, Spaak P 2016 Sedimentary DNA reveals cyanobacterial community diversity over 200 years in two perialpine lakes. Appl Environ Microbiol 82:6472–6482. doi:10.1128/AEM.02174-16. 27565621 PMC5066364

[20] Monchamp M-E, Spaak P, Pomati F 2019 High dispersal levels and lake warming are emergent drivers of cyanobacterial community assembly in peri-Alpine lakes. Sci Rep 9:7366. doi:10.1038/s41598-019-43814-2. 31089175 PMC6517590

[21] Pilon S, Zastepa A, Taranu ZE, Gregory-Eaves I, Racine M, Blais JM, Poulain AJ, Pick FR 2019 Contrasting histories of microcystin-producing cyanobacteria in two temperate lakes as inferred from quantitative sediment DNA analyses. Lake Reserv Manag 35:102–117. doi:10.1080/10402381.2018.1549625.

[22] Capo E, Debroas D, Arnaud F, Guillemot T, Bichet V, Millet L, Gauthier E, Massa C, Develle AL, Pignol C, Lejzerowicz F, Domaizon I 2016 Long-term dynamics in microbial eukaryotes communities: a palaeolimnological view based on sedimentary DNA. Mol Ecol 25:5925–5943. doi:10.1111/mec.13893. 27761959

[23] Capo E, Debroas D, Arnaud F, Perga ME, Chardon C, Domaizon I 2017 Tracking a century of changes in microbial eukaryotic diversity in lakes driven by nutrient enrichment and climate warming. Environ Microbiol 19:2873–2892. doi:10.1111/1462-2920.13815. 28585365

[24] Poulain AJ, Aris-Brosou S, Blais JM, Brazeau M, Keller W, Paterson AM 2015 Microbial DNA records historical delivery of anthropogenic mercury. ISME J 9:2541–2550. doi:10.1038/ismej.2015.86. 26057844 PMC4817628

[25] Belle S, Parent C 2019 Reconstruction of past dynamics of methane-oxidizing bacteria in lake sediments using a quantitative PCR method: connecting past environmental changes and microbial community. Geomicrobiol J 36:570–579. doi:10.1080/01490451.2019.1583698.

[26] Eloe-Fadrosh EA, Ivanova NN, Woyke T, Kyrpides NC 2016 Metagenomics uncovers gaps in amplicon-based detection of microbial diversity. Nat Microbiol 1:15032. doi:10.1038/nmicrobiol.2015.32. 27572438

[27] Piwosz K, Shabarova T, Pernthaler J, Posch T, Šimek K, Porcal P, Salcher MM 2020 Bacterial and eukaryotic small-subunit amplicon data do not provide a quantitative picture of microbial communities, but they are reliable in the context of ecological interpretations. mSphere 5:e00052-20. doi:10.1128/mSphere.00052-20. 32132159 PMC7056804

[28] Taberlet P, Coissac E, Pompanon F, Brochmann C, Willerslev E 2012 Towards next-generation biodiversity assessment using DNA metabarcoding. Mol Ecol 21:2045–2050. doi:10.1111/j.1365-294X.2012.05470.x. 22486824

[29] Ziesemer KA, Mann AE, Sankaranarayanan K, Schroeder H, Ozga AT, Brandt BW, Zaura E, Waters-Rist A, Hoogland M, Salazar-García DC, Aldenderfer M, Speller C, Hendy J, Weston DA, MacDonald SJ, Thomas GH, Collins MJ, Lewis CM, Hofman C, Warinner C 2015 Intrinsic challenges in ancient microbiome reconstruction using 16S rRNA gene amplification. Sci Rep 5:16498. doi:10.1038/srep16498. 26563586 PMC4643231

[30] Pedersen MW, Ruter A, Schweger C, Friebe H, Staff RA, Kjeldsen KK, Mendoza MLZ, Beaudoin AB, Zutter C, Larsen NK, Potter BA, Nielsen R, Rainville RA, Orlando L, Meltzer DJ, Kjær KH, Willerslev E 2016 Postglacial viability and colonization in North America’s ice-free corridor. Nature 537:45–49. doi:10.1038/nature19085. 27509852

[31] Parducci L, Alsos IG, Unneberg P, Pedersen MW, Han L, Lammers Y, Salonen JS, Väliranta MM, Slotte T, Wohlfarth B 2019 Shotgun environmental DNA, pollen, and macrofossil analysis of lateglacial lake sediments from southern Sweden. Front Ecol Evol 7:189. doi:10.3389/fevo.2019.00189.

[32] Schulte L, Bernhardt N, Stoof-Leichsenring KR, Zimmermann HH, Pestryakova LA, Epp LS, Herzschuch U 2020 Hybridization capture of larch (Larix Mill) chloroplast genomes from sedimentary ancient DNA reveals past changes of Siberian forests 1–21. bioRxiv doi:10.1101/2020.01.06.896068. 33319428

[33] Lammers Y, Heintzman PD, Alsos IG 2020 Environmental palaeogenomic reconstruction of an Ice Age algal population. bioRxiv doi:10.1101/2020.04.10.035535. PMC7887274 33594237

[34] Ahmed E, Parducci L, Unneberg P, Ågren R, Schenk F, Rattray JE, Han L, Muschitiello F, Pedersen MW, Smittenberg RH, Yamoah KA, Slotte T, Wohlfarth B 2018 Archaeal community changes in Lateglacial lake sediments: evidence from ancient DNA. Quat Sci Rev 181:19–29. doi:10.1016/j.quascirev.2017.11.037.

[35] Torti A, Jørgensen BB, Lever MA 2018 Preservation of microbial DNA in marine sediments: insights from extracellular DNA pools. Environ Microbiol 20:4526–4542. doi:10.1111/1462-2920.14401. 30198168

[36] Coenye T 2014 The family Burkholderiaceae, p 759–776. *In* Rosenberg E, DeLong EF, Lory S, Stackebrandt E, Thompson F (ed), The prokaryotes: Alphaproteobacteria and Betaproteobacteria. Springer, Berlin, Germany.

[37] Willems A 2014 The family Comamonadaceae, p 777–851. *In* Rosenberg E, DeLong EF, Lory S, Stackebrandt E, Thompson F (ed), The prokaryotes: Alphaproteobacteria and Betaproteobacteria. Springer, Berlin, Germany.

[38] Newton RJ, Jones SE, Eiler A, McMahon KD, Bertilsson S 2011 A guide to the natural history of freshwater lake bacteria. Microbiol Mol Biol Rev 75:14–49. doi:10.1128/MMBR.00028-10. 21372319 PMC3063352

[39] Salcher MM, Pernthaler J, Posch T 2011 Seasonal bloom dynamics and ecophysiology of the freshwater sister clade of SAR11 bacteria ‘that rule the waves’ (LD12). ISME J 5:1242–1252. doi:10.1038/ismej.2011.8. 21412347 PMC3146277

[40] Neuenschwander SM, Ghai R, Pernthaler J, Salcher MM 2018 Microdiversification in genome-streamlined ubiquitous freshwater Actinobacteria. ISME J 12:185–198. doi:10.1038/ismej.2017.156. 29027997 PMC5739012

[41] Henson MW, Lanclos VC, Faircloth BC, Thrash JC 2018 Cultivation and genomics of the first freshwater SAR11 (LD12) isolate. ISME J 12:1846–1860. doi:10.1038/s41396-018-0092-2. 29599519 PMC6018831

[42] Salcher MM, Schaefle D, Kaspar M, Neuenschwander SM, Ghai R 2019 Evolution in action: habitat transition from sediment to the pelagial leads to genome streamlining in Methylophilaceae. ISME J 13:2764–2777. doi:10.1038/s41396-019-0471-3. 31292537 PMC6794327

[43] Boere AC, Sinninghe Damsté JS, Rijpstra WIC, Volkman JK, Coolen MJL 2011 Source-specific variability in post-depositional DNA preservation with potential implications for DNA based paleoecological records. Org Geochem 42:1216–1225. doi:10.1016/j.orggeochem.2011.08.005.

[44] Capo E, Debroas D, Arnaud F, Domaizon I 2015 Is planktonic diversity well recorded in sedimentary DNA? Toward the reconstruction of past protistan diversity. Microb Ecol 70:865–875. doi:10.1007/s00248-015-0627-2. 26022714

[45] Vuillemin A, Horn F, Alawi M, Henny C, Wagner D, Crowe SA, Kallmeyer J 2017 Preservation and significance of extracellular DNA in ferruginous sediments from Lake Towuti, Indonesia. Front Microbiol 8:1440. doi:10.3389/fmicb.2017.01440. 28798742 PMC5529349

[46] Maniloff J, Ackermann H-W 1998 Taxonomy of bacterial viruses: establishment of tailed virus genera and the other Caudovirales. Arch Virol 143:2051–2063. doi:10.1007/s007050050442. 9856093

[47] Canchaya C, Proux C, Fournous G, Bruttin A, Brüssow H 2003 Prophage genomics. Microbiol Mol Biol Rev 67:238–276. doi:10.1128/MMBR.67.3.473.2003. 12794192 PMC156470

[48] Coolen MJL 2011 7000 years of *Emiliania huxleyi* viruses in the Black Sea. Science 333:451–452. doi:10.1126/science.1200072. 21778399

[49] Ng TFF, Chen L-F, Zhou Y, Shapiro B, Stiller M, Heintzman PD, Varsani A, Kondov NO, Wong W, Deng X, Andrews TD, Moorman BJ, Meulendyk T, MacKay G, Gilbertson RL, Delwart E 2014 Preservation of viral genomes in 700-y-old caribou feces from a subarctic ice patch. Proc Natl Acad Sci U S A 111:16842–16847. doi:10.1073/pnas.1410429111. 25349412 PMC4250163

[50] Fuhrman JA 1999 Marine viruses and their biogeochemical and ecological effects. Nature 399:541–548. doi:10.1038/21119. 10376593

[51] Kavagutti VS, Andrei A-Ş, Mehrshad M, Salcher MM, Ghai R 2019 Phage-centric ecological interactions in aquatic ecosystems revealed through ultra-deep metagenomics. Microbiome 7:135. doi:10.1186/s40168-019-0752-0. 31630686 PMC6802176

[52] Pernthaler J 2017 Competition and niche separation of pelagic bacteria in freshwater habitats. Environ Microbiol 19:2133–2150. doi:10.1111/1462-2920.13742. 28370850

[53] Rocap G, Larimer FW, Lamerdin J, Malfatti S, Chain P, Ahlgren NA, Arellano A, Coleman M, Hauser L, Hess WR, Johnson ZI, Land M, Lindell D, Post AF, Regala W, Shah M, Shaw SL, Steglich C, Sullivan MB, Ting CS, Tolonen A, Webb EA, Zinser ER, Chisholm SW 2003 Genome divergence in two *Prochlorococcus* ecotypes reflects oceanic niche differentiation. Nature 424:1042–1047. doi:10.1038/nature01947. 12917642

[54] Cohan FM 2006 Towards a conceptual and operational union of bacterial systematics, ecology, and evolution. Philos Trans R Soc Lond B Biol Sci 361:1985–1996. doi:10.1098/rstb.2006.1918. 17062416 PMC1764936

[55] Hahn MW, Jezberová J, Koll U, Saueressig-Beck T, Schmidt J 2016 Complete ecological isolation and cryptic diversity in *Polynucleobacter* bacteria not resolved by 16S rRNA gene sequences. ISME J 10:1642–1655. doi:10.1038/ismej.2015.237. 26943621 PMC4913878

[56] Hahn MW, Schmidt J, Pitt A, Taipale SJ, Lang E 2016 Reclassification of four *Polynucleobacter necessarius* strains as representatives of *Polynucleobacter asymbioticus* comb. nov., *Polynucleobacter duraquae* sp. nov., *Polynucleobacter yangtzensis* sp. nov. and *Polynucleobacter sinensis* sp. nov., and emended description of *Polynucleobacter necessarius*. Int J Syst Evol Microbiol 66:2883–2892. doi:10.1099/ijsem.0.001073. 27064460 PMC5018217

[57] Props R, Denef VJ 2020 Temperature and nutrient levels correspond with lineage-specific microdiversification in the ubiquitous and abundant freshwater genus *Limnohabitans*. Appl Environ Microbiol 86:e00140-20. doi:10.1128/AEM.00140-20. 32169939 PMC7205491

[58] Nuy JK, Hoetzinger M, Hahn MW, Beisser D, Boenigk J 2020 Ecological differentiation in two major freshwater bacterial taxa along environmental gradients. Front Microbiol 11:154. doi:10.3389/fmicb.2020.00154. 32117171 PMC7031163

[59] Huot Y, Brown CA, Potvin G, Antoniades D, Baulch HM, Beisner BE, Bélanger S, Brazeau S, Cabana H, Cardille JA, del Giorgio PA, Gregory-Eaves I, Fortin M-J, Lang AS, Laurion I, Maranger R, Prairie YT, Rusak JA, Segura PA, Siron R, Smol JP, Vinebrooke RD, Walsh DA 2019 The NSERC Canadian Lake Pulse Network: a national assessment of lake health providing science for water management in a changing climate. Sci Total Environ 695:133668. doi:10.1016/j.scitotenv.2019.133668. 31419692

[60] Melton ED, Stief P, Behrens S, Kappler A, Schmidt C 2014 High spatial resolution of distribution and interconnections between Fe- and N-redox processes in profundal lake sediments. Environ Microbiol 16:3287–3303. doi:10.1111/1462-2920.12566. 25041287

[61] Walsh EA, Kirkpatrick JB, Rutherford SD, Smith DC, Sogin M, D’Hondt S 2016 Bacterial diversity and community composition from seasurface to subseafloor. ISME J 10:979–989. doi:10.1038/ismej.2015.175. 26430855 PMC4796937

[62] Orsi WD, Coolen MJL, Wuchter C, He L, More KD, Irigoien X, Chust G, Johnson C, Hemingway JD, Lee M, Galy V, Giosan L 2017 Climate oscillations reflected within the microbiome of Arabian Sea sediments. Sci Rep 7:6040. doi:10.1038/s41598-017-05590-9. 28729646 PMC5519670

[63] More KD, Giosan L, Grice K, Coolen MJL 2019 Holocene paleodepositional changes reflected in the sedimentary microbiome of the Black Sea. Geobiology 17:436–448. doi:10.1111/gbi.12338. 30843322

[64] Schelske CL, Peplow A, Brenner M, Spencer CN 1994 Low-background gamma counting: applications for 210Pb dating of sediments. J Paleolimnol 10:115–128. doi:10.1007/BF00682508.

[65] Bolger AM, Lohse M, Usadel B 2014 Trimmomatic: a flexible trimmer for Illumina sequence data. Bioinformatics 30:2114–2120. doi:10.1093/bioinformatics/btu170. 24695404 PMC4103590

[66] Li D, Luo R, Liu C-M, Leung C-M, Ting H-F, Sadakane K, Yamashita H, Lam T-W 2016 MEGAHIT v1.0: a fast and scalable metagenome assembler driven by advanced methodologies and community practices. Methods 102:3–11. doi:10.1016/j.ymeth.2016.02.020. 27012178

[67] Huntemann M, Ivanova NN, Mavromatis K, Tripp HJ, Paez-Espino D, Tennessen K, Palaniappan K, Szeto E, Pillay M, Chen I-MA, Pati A, Nielsen T, Markowitz VM, Kyrpides NC 2016 The standard operating procedure of the DOE-JGI Metagenome Annotation Pipeline (MAP v.4). Stand Genomic Sci 11:17. doi:10.1186/s40793-016-0138-x. 26918089 PMC4766715

[68] Bushnell B 2015 BBMap. https://sourceforge.net/projects/bbmap/.

[69] Quinlan AR, Hall IM 2010 BEDTools: a flexible suite of utilities for comparing genomic features. Bioinformatics 26:841–842. doi:10.1093/bioinformatics/btq033. 20110278 PMC2832824

[70] Oksanen J, Blanchet FG, Kindt R, Legen P, Minchin PR, Hara RBO, Simpson GL, Soly P, Stevens MHH, Wagner H 2019 vegan: community ecology package. R package version 25-4.

[71] R Core Team. 2018 R: a language and environment for statistical computing. R Foundation for Statistical Computing, Vienna, Austria.

[72] Wickham H, Averick M, Bryan J, Chang W, McGowan L, François R, Grolemund G, Hayes A, Henry L, Hester J, Kuhn M, Pedersen T, Miller E, Bache S, Müller K, Ooms J, Robinson D, Seidel D, Spinu V, Takahashi K, Vaughan D, Wilke C, Woo K, Yutani H 2019 Welcome to the Tidyverse. J Open Source Softw 4:1686. doi:10.21105/joss.01686.

[73] Roux S, Enault F, Robin A, Ravet V, Personnic S, Theil S, Colombet J, Sime-Ngando T, Debroas D 2012 Assessing the diversity and specificity of two freshwater viral communities through metagenomics. PLoS One 7:e33641. doi:10.1371/journal.pone.0033641. 22432038 PMC3303852

[74] Katoh K, Rozewicki J, Yamada KD 2019 MAFFT online service: multiple sequence alignment, interactive sequence choice and visualization. Brief Bioinform 20:1160–1166. doi:10.1093/bib/bbx108. 28968734 PMC6781576

[75] Jones DT, Taylor WR, Thornton JM 1992 The rapid generation of mutation data matrices from protein sequences. Comput Appl Biosci 8:275–282. doi:10.1093/bioinformatics/8.3.275. 1633570

[76] Kumar S, Stecher G, Li M, Knyaz C, Tamura K 2018 MEGA X: Molecular Evolutionary Genetics Analysis across computing platforms. Mol Biol Evol 35:1547–1549. doi:10.1093/molbev/msy096. 29722887 PMC5967553

[77] QGIS Geographic Information System. 2020 Open Source Geospatial Foundation Project.

[78] Natural Resources Canada. 2019 Lakes, rivers and glaciers in Canada: CanVec series hydrographic features. Natural Resources Canada. http://ftp.maps.canada.ca/pub/nrcan_rncan/vector/canvec/shp/Hydro/canvec_1M_CA_Hydro_shp.zip. Accessed 22 May 2020.

[79] Statistics Canada. 2019 Boundary files, 2011 census. Stats Canada catalog no 92-160-X. Statistics Canada, Ottawa, Ontario, Canada. https://www12.statcan.gc.ca/census-recensement/2011/geo/bound-limit/bound-limit-2011-eng.cfm. Accessed 22 May 2020.

