# Supplementary figures and images for: Sediment Metagenomes as Time Capsules of Lake Microbiomes
Source: mSphere. 2020 Nov 4;5(6):e00512-20. doi: 10.1128/mSphere.00512-20 (PMC7643826; doi:10.1128/mSphere.00512-20)

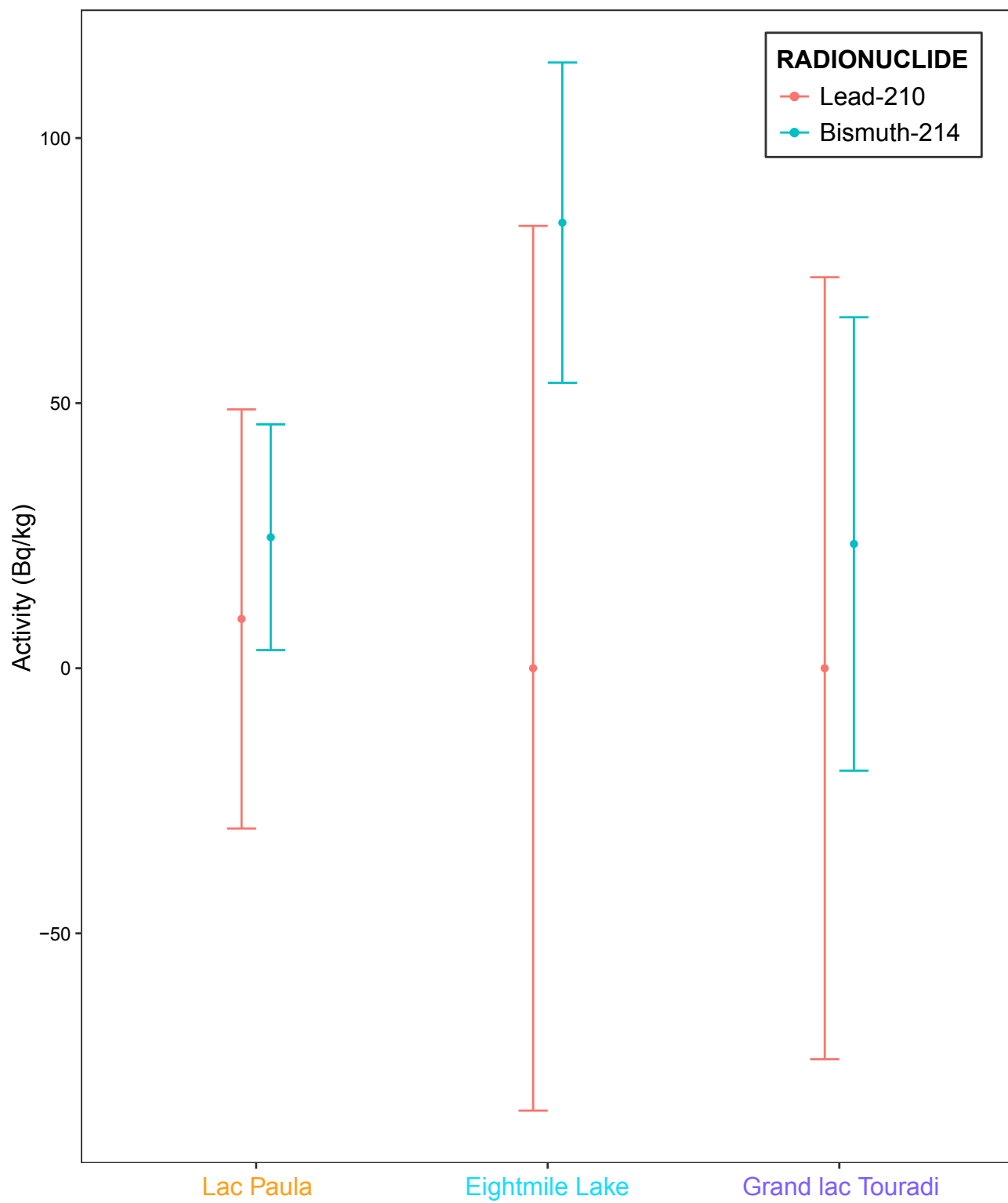

Supplement: FIG S1 [file mSphere.00512-20-sf001.pdf]

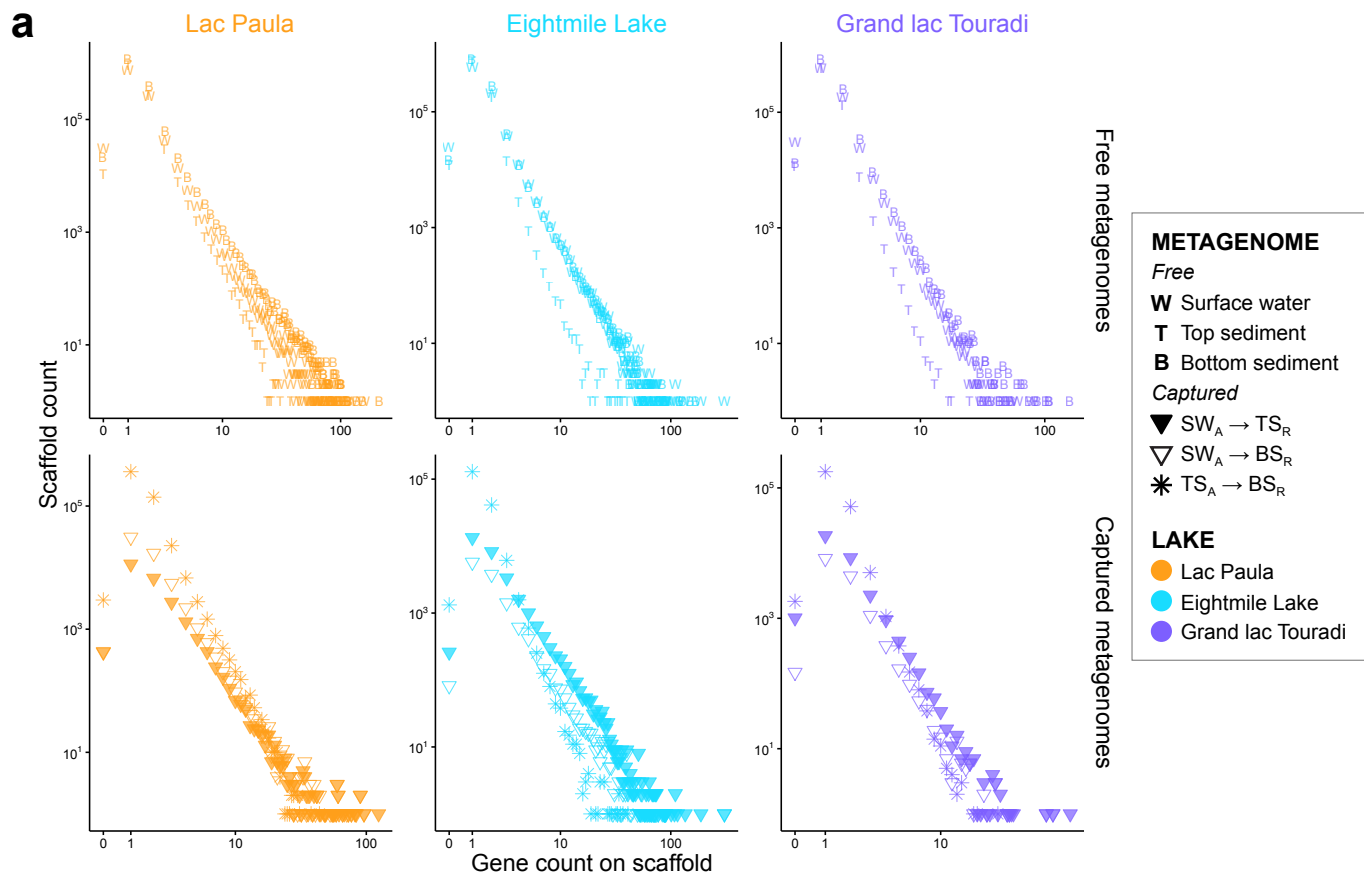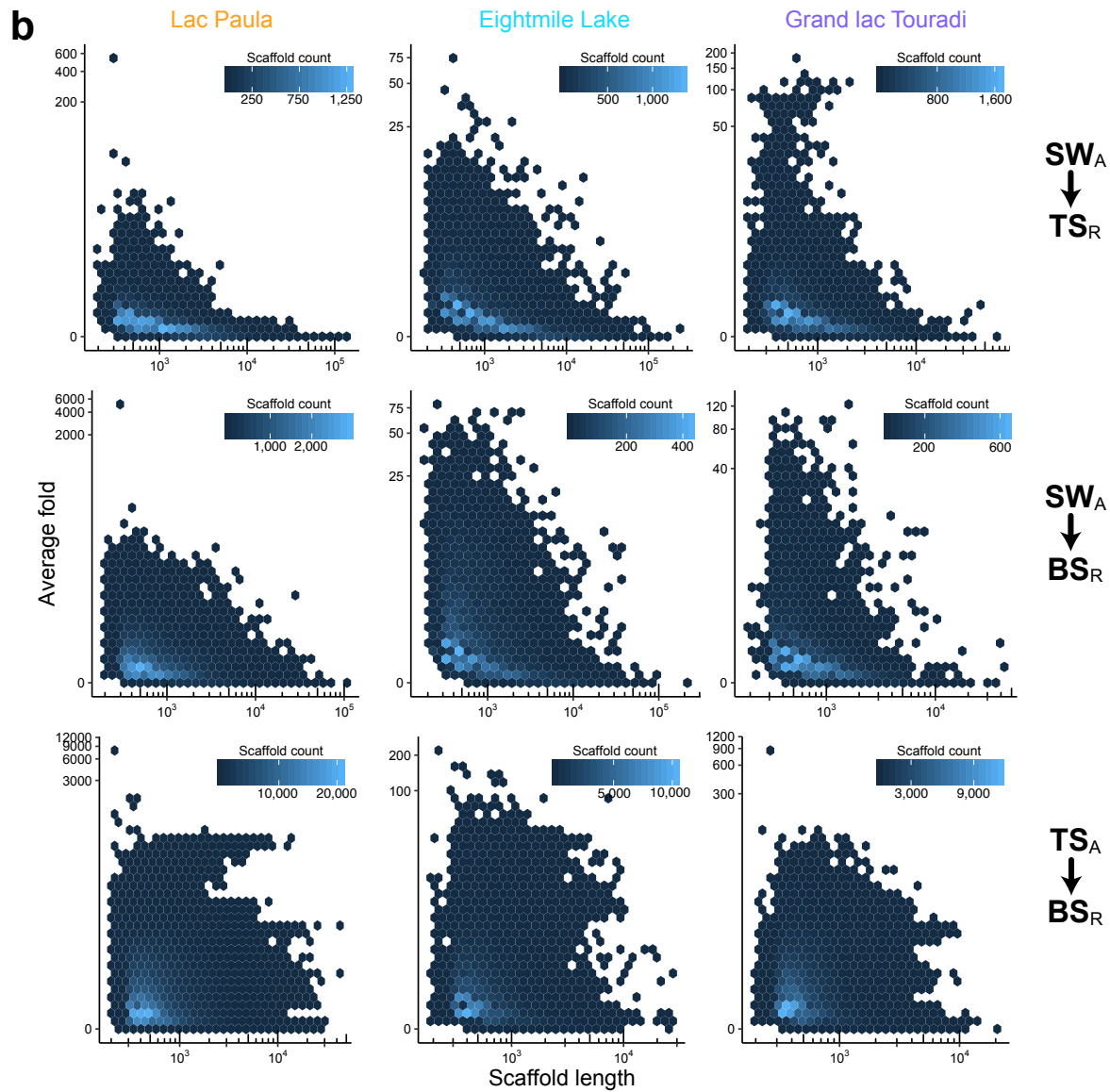

Supplement: FIG S2 [file mSphere.00512-20-sf002.pdf]

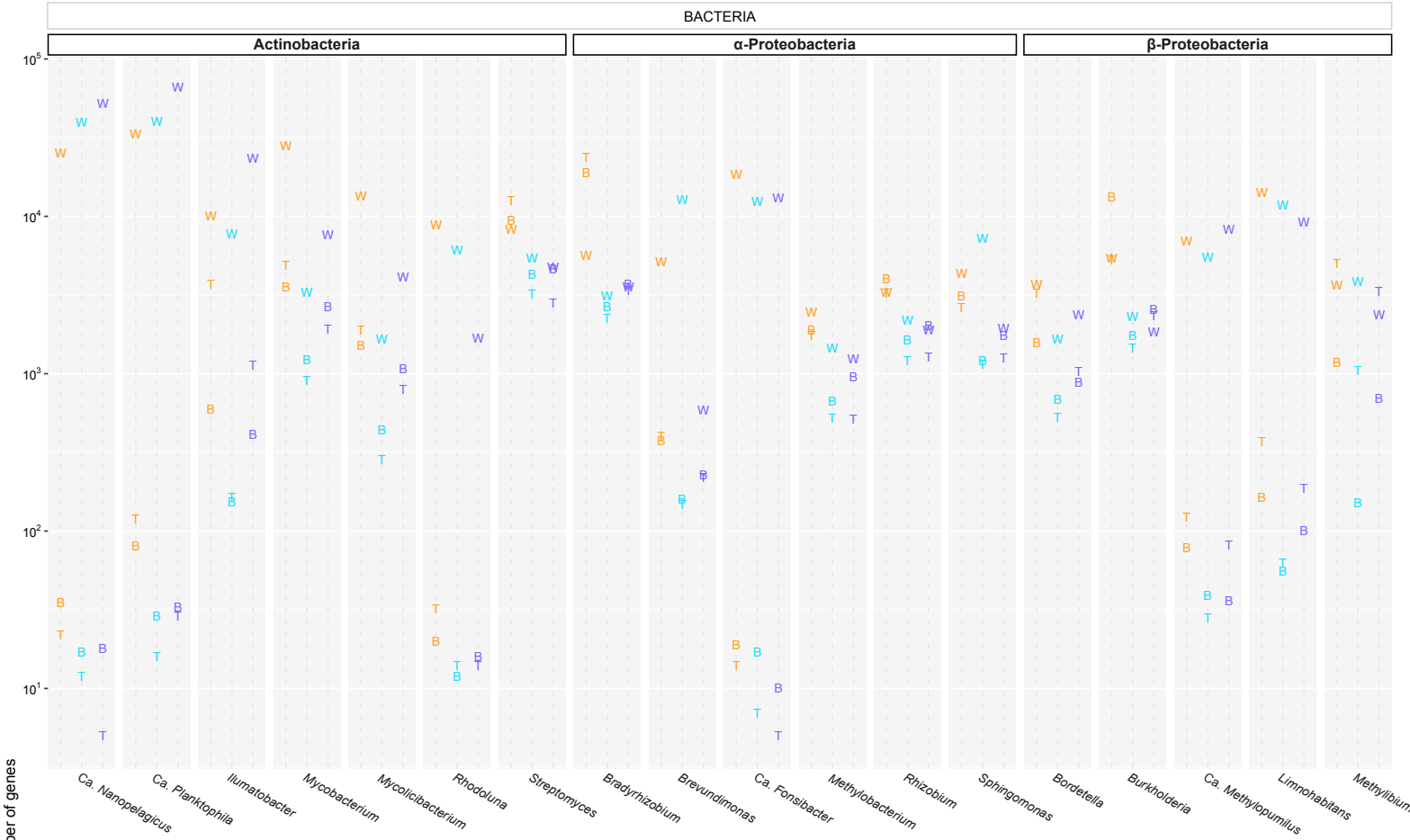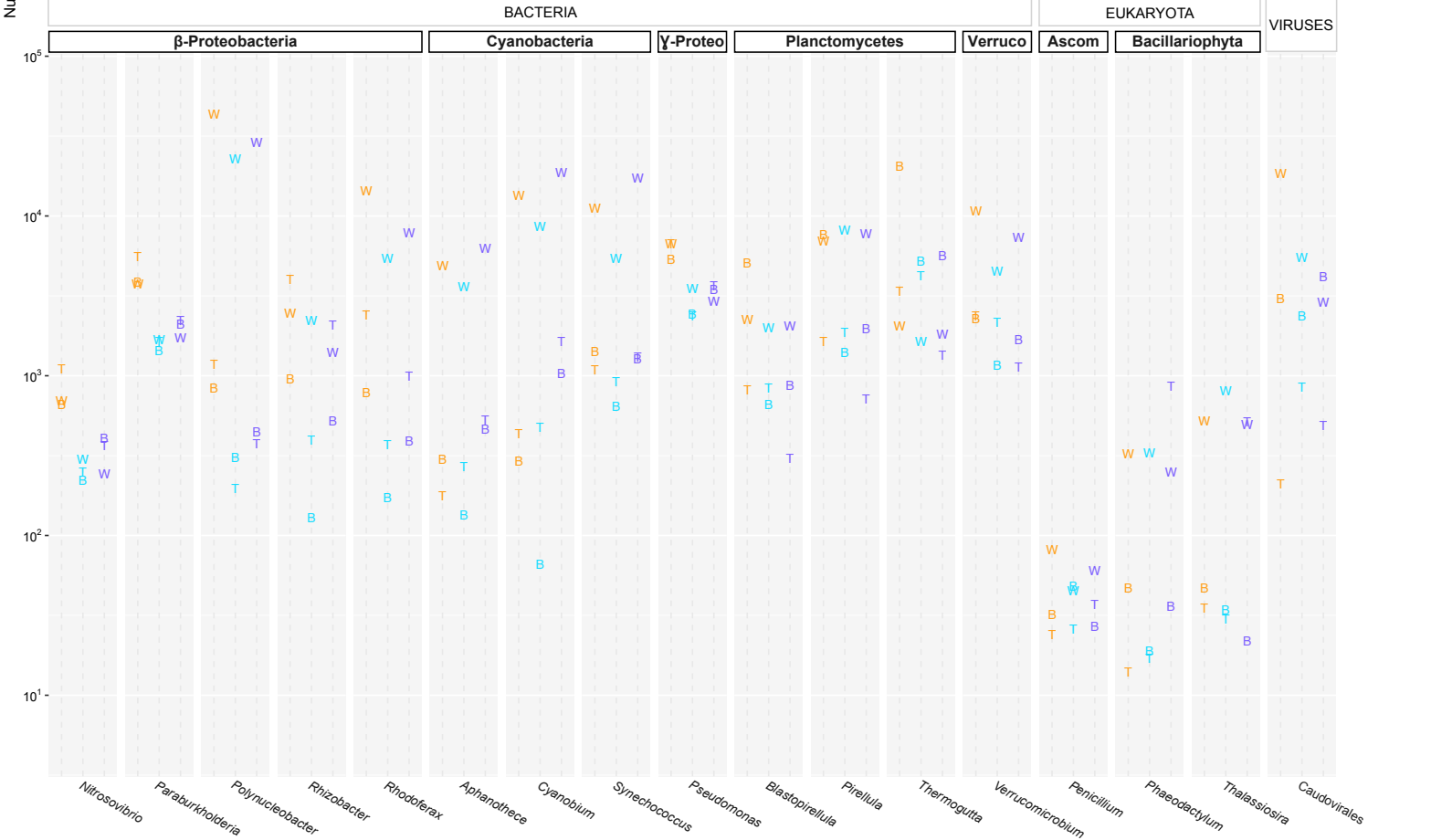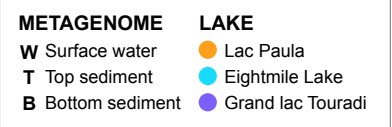

Supplement: FIG S4 [file mSphere.00512-20-sf004.pdf]

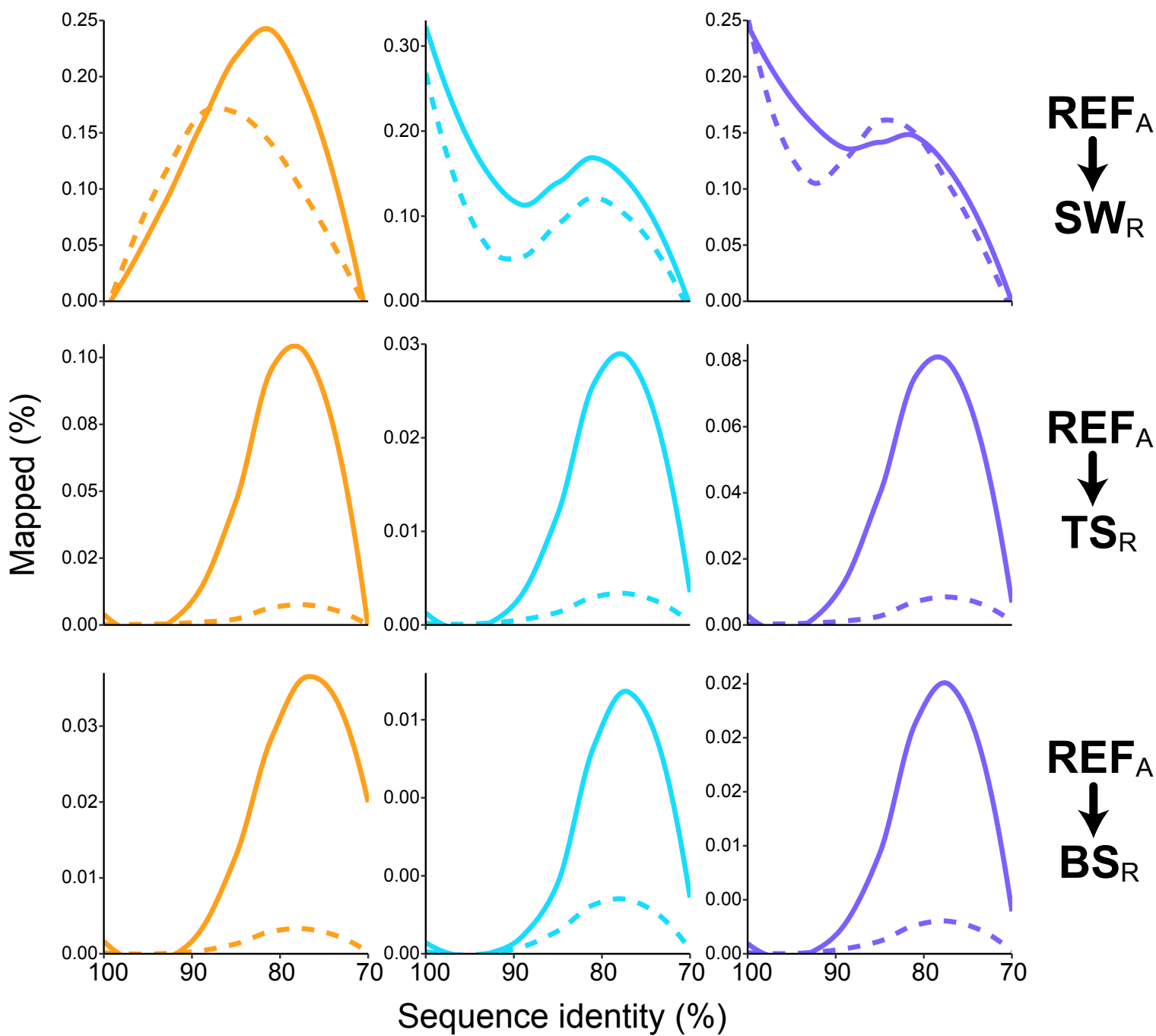

Supplement: FIG S5 [file mSphere.00512-20-sf005.pdf]
