# Supplementary material for: Sediment Metagenomes as Time Capsules of Lake Microbiomes
Source: mSphere. 2020 Nov 4;5(6):e00512-20. doi: 10.1128/mSphere.00512-20 (PMC7643826; doi:10.1128/mSphere.00512-20)
Supplement: TABLE S1 [file mSphere.00512-20-st001.pdf]

Table S1. Comparisons of the number of scaffolds and mapped reads in captured metagenomes before and after scaffolds containing ribosomal or transfer RNA genes were removed.

Lake	Metagenome	All scaffolds		Scaffolds *not* containing r/tRNA genes	
		**Scaffold count**	**Mapped read count**	**Scaffold count**	**Mapped read count**
Lac Paula	$SW_A \rightarrow TS_R$	25,477	80,595	24,627	69,318
	$SW_A \rightarrow BS_R$	60,151	525,474	59,216	511,831
	$TS_A \rightarrow BS_R$	543,910	14,669,611	539,670	14,234,026
Eightmile Lake	$SW_A \rightarrow TS_R$	31,767	153,170	30,749	145,692
	$SW_A \rightarrow BS_R$	13,382	190,077	13,067	188,125
	$TS_A \rightarrow BS_R$	182,822	1,669,845	180,586	1,622,267
Grand lac Touradi	$SW_A \rightarrow TS_R$	33,103	155,485	32,284	143,854
	$SW_A \rightarrow BS_R$	15,196	101,450	14,772	97,815
	$TS_A \rightarrow BS_R$	239,592	2,368,008	237,551	2,324,829
