# Supplementary material for: Sediment Metagenomes as Time Capsules of Lake Microbiomes
Source: mSphere. 2020 Nov 4;5(6):e00512-20. doi: 10.1128/mSphere.00512-20 (PMC7643826; doi:10.1128/mSphere.00512-20)
Supplement: TABLE S2 [file mSphere.00512-20-st002.pdf]

Table S2. Relative coverage of phyla in the free and captured metagenomes of three lakes.

Metagenome type	Metagenome	Phylum	Relative coverage (%)		
			Lac Paula	Eightmile Lake	Grand lac Touradi
free	SW_A	Acetothermia	0.0	0.0	0.0
		Acidithiobacillia	0.0	0.0	0.0
		Acidobacteria	0.3	0.2	0.2
		Actinobacteria	27.3	31.5	49.7
		Aerophobetes	0.0	0.0	0.0
		Aigarchaeota	0.0	0.0	0.0
		Alphaproteobacteria	19.8	20.6	13.6
		Aminicenantes	0.0	0.0	0.0
		Annelida	0.0	0.0	0.0
		Apicomplexa	0.1	0.1	0.0
		Aquificae	0.0	0.0	0.0
		Armatimonadetes	0.3	0.2	0.2
		Arthropoda	0.1	0.2	0.2
		Ascomycota	0.2	0.2	0.1
		Atribacteria	0.0	0.0	0.0
		Bacillariophyta	0.0	0.1	0.1
		Bacteroidetes	5.3	10.4	3.7
		Balneolaeota	0.0	0.0	0.0
		Basidiomycota	0.1	0.1	0.1
		Betaproteobacteria	20.7	16.1	13.8
		Blastocladiomycota	0.0	0.0	0.0
		BRC1	0.0	0.0	0.0
		Caldiserica	0.0	0.0	0.0
		Caldithrixae	0.0	0.0	0.0
		Calescamantes	0.0	0.0	0.0
		Candidatus Atribacteria	0.0	0.0	0.0
		Candidatus Bipolaricaulota	0.0	0.0	0.0
		Candidatus Korarchaeota	0.0	0.0	0.0
		Candidatus Melainabacteria	0.0	0.1	0.0
		Candidatus Micrarchaeota	0.0	0.0	NA
		Candidatus Microgenomates	0.0	0.0	0.0
		Candidatus Poribacteria	0.0	0.0	0.0
		Candidatus Saccharibacteria	0.1	0.0	0.0
		Chlamydiae	0.1	0.2	0.0
		Chlorobi	0.1	0.1	0.1
		Chloroflexi	0.6	0.6	0.6
		Chlorophyta	0.1	0.1	0.1
		Chordata	0.1	0.1	0.1
		Chrysiogenetes	0.0	0.0	0.0
		Chytridiomycota	0.1	0.1	0.0
		Cloacimonetes	0.0	0.0	0.0
		Cnidaria	0.0	0.0	0.0
		Coprothermobacterota	0.0	0.0	0.0
		Crenarchaeota	0.0	0.0	0.0
		Cyanobacteria	4.0	3.3	4.0
		Deferribacteres	0.0	0.0	0.0
		Deinococcus-Thermus	0.1	0.1	0.1
		Deltaproteobacteria	1.0	1.6	0.6
		Diapherotrites	0.0	0.0	0.0
		Dictyoglomi	0.0	0.0	0.0
		Elusimicrobia	0.0	0.0	0.0
		EM3	0.0	NA	0.0

		Epsilonproteobacteria	0.1	0.1	0.1
		Euryarchaeota	0.1	0.1	0.1
		Fervidibacteria	0.0	0.0	0.0
		Fibrobacteres	0.0	0.0	0.0
		Firmicutes	1.7	1.2	0.9
		Fusobacteria	0.0	0.0	0.0
		Gammaproteobacteria	3.1	2.4	2.0
		Gemmatimonadetes	0.1	0.0	0.4
		Gracilibacteria	0.0	0.0	0.0
		Hydrogenedentes	0.0	0.0	0.0
		Hydrogenophilalia	0.0	0.0	0.0
		Ignavibacteriae	0.0	0.0	0.0
		Latescibacteria	0.0	0.0	0.0
		Lentisphaerae	0.0	0.0	0.0
		Marinimicrobia	0.0	0.0	0.0
		Microgenomates	0.0	0.0	0.0
		Microsporidia	0.0	0.0	0.0
		Mollusca	0.0	0.0	0.0
		Mucoromycota	0.0	0.0	0.0
		Nanoarchaeota	0.0	0.0	0.0
		Nematoda	0.0	0.0	0.0
		Nitrospinae	0.0	0.0	0.0
		Nitrospirae	0.1	0.1	0.1
		Oligoflexia	0.2	0.2	0.1
		Omnitrophica	0.0	0.0	0.0
		Parcubacteria	0.0	0.1	0.0
		PER	0.0	0.0	0.0
		Placozoa	0.0	0.0	0.0
		Planctomycetes	2.3	2.2	1.7
		Poribacteria	0.0	0.0	0.0
		Porifera	0.0	0.1	0.0
		Rhodothermaeota	0.0	0.0	0.0
		Spirochaetes	0.2	0.1	0.1
		Streptophyta	0.1	0.1	0.1
		Synergistetes	0.0	0.0	0.0
		Tenericutes	0.0	0.0	0.0
		Thaumarchaeota	0.0	0.0	0.0
		Thermodesulfobacteria	0.0	0.0	0.0
		Thermotogae	0.0	0.0	0.0
		unclassified Bacteria	0.0	0.0	0.0
		unclassified Eukaryota	0.3	0.3	0.2
		unclassified Proteobacteria	0.0	0.0	NA
		Verrucomicrobia	6.8	3.7	5.8
		Viruses	3.6	2.6	0.8
		WS1	0.0	0.0	0.0
		Zetaproteobacteria	0.0	0.0	0.0
		Zoopagomycota	0.0	0.0	0.0
	TS_A	Acetothermia	0.0	0.1	0.0
		Acidithiobacillia	0.1	0.1	0.1
		Acidobacteria	5.0	1.9	2.9
		Actinobacteria	10.1	2.6	3.5
		Aerophobetes	0.1	0.4	0.2
		Aigarchaeota	0.0	0.1	0.0
		Alphaproteobacteria	9.3	3.1	4.6
		Aminicenantes	0.6	1.0	1.5
		Annelida	0.0	0.0	0.0

		Apicomplexa	0.0	0.0	0.0
		Aquificae	0.2	0.2	0.2
		Armatimonadetes	0.2	0.3	0.1
		Arthropoda	0.0	0.0	0.0
		Ascomycota	0.0	0.1	0.1
		Atribacteria	0.1	0.3	0.2
		Bacillariophyta	0.0	0.0	0.3
		Bacteroidetes	2.3	6.5	3.3
		Balneolaeota	0.1	0.1	0.1
		Basidiomycota	0.0	0.0	0.1
		Betaproteobacteria	14.0	3.8	15.4
		Blastocladiomycota	0.0	0.0	0.0
		BRC1	0.1	0.4	0.1
		Caldiserica	0.0	0.0	0.0
		Caldithrixae	0.2	0.5	0.2
		Calescamantes	0.0	0.0	0.0
		Candidatus Atribacteria	0.1	0.1	0.1
		Candidatus Bipolaricaulota	0.0	0.0	0.0
		Candidatus Korarchaeota	0.0	0.0	0.0
		Candidatus Melainabacteria	0.0	0.0	0.0
		Candidatus Micrarchaeota	0.0	0.0	0.0
		Candidatus Microgenomates	0.0	0.0	0.0
		Candidatus Poribacteria	0.1	0.1	0.1
		Candidatus Saccharibacteria	0.0	0.0	0.0
		Chlamydiae	0.1	0.1	0.0
		Chlorobi	0.3	0.5	0.3
		Chloroflexi	4.9	7.6	3.1
		Chlorophyta	0.0	0.0	0.0
		Chordata	0.0	0.0	0.0
		Chrysiogenetes	0.0	0.0	0.0
		Chytridiomycota	0.0	0.0	0.0
		Cloacimonetes	0.1	0.3	0.1
		Cnidaria	0.0	0.0	0.0
		Coprothermobacterota	0.0	0.0	0.0
		Crenarchaeota	0.1	0.3	0.1
		Cyanobacteria	1.8	2.3	2.0
		Deferribacteres	0.1	0.1	0.1
		Deinococcus-Thermus	0.5	0.3	0.2
		Deltaproteobacteria	20.3	18.9	21.7
		Diapherotrites	0.0	0.1	0.0
		Dictyoglomi	0.0	0.1	0.0
		Elusimicrobia	0.0	0.1	0.0
		EM3	0.0	0.0	0.0
		Epsilonproteobacteria	0.1	0.1	0.1
		Euryarchaeota	1.5	15.7	8.1
		Fervidibacteria	0.0	0.1	0.0
		Fibrobacteres	0.0	0.2	0.1
		Firmicutes	6.4	9.3	5.2
		Fusobacteria	0.0	0.1	0.0
		Gammaproteobacteria	8.4	7.0	11.2
		Gemmatimonadetes	1.4	0.4	1.0
		Gracilibacteria	0.0	0.0	0.0
		Hydrogenedentes	0.0	0.1	0.1
		Hydrogenophilalia	0.0	0.0	0.0
		Ignavibacteriae	0.3	0.9	0.7
		Latescibacteria	0.2	0.4	0.2

		Lentisphaerae	0.1	0.3	0.1
		Marinimicrobia	0.1	0.3	0.1
		Microgenomates	0.0	0.0	0.0
		Microsporidia	0.0	0.0	0.0
		Mollusca	0.0	0.0	0.0
		Mucoromycota	0.0	0.0	0.0
		Nanoarchaeota	0.0	0.0	0.0
		Nematoda	0.0	0.0	0.0
		Nitrospinae	0.1	0.1	0.1
		Nitrospirae	3.1	0.8	5.4
		Oligoflexia	0.2	0.1	0.1
		Omnitrophica	0.1	0.6	0.1
		Parcubacteria	0.0	0.1	0.0
		PER	0.0	0.0	0.0
		Placozoa	0.0	0.0	0.0
		Planctomycetes	2.9	4.3	2.0
		Poribacteria	0.0	0.0	0.0
		Porifera	0.0	0.0	0.0
		Rhodothermaeota	0.1	0.1	0.1
		Spirochaetes	0.7	1.4	1.2
		Streptophyta	0.0	0.0	0.1
		Synergistetes	0.2	0.2	0.2
		Tenericutes	0.0	0.0	0.0
		Thaumarchaeota	0.1	0.2	0.1
		Thermodesulfobacteria	0.3	0.2	0.2
		Thermotogae	0.1	0.3	0.1
		unclassified Bacteria	0.1	0.1	0.1
		unclassified Eukaryota	0.0	0.1	0.1
		unclassified Proteobacteria	NA	0.0	NA
		Verrucomicrobia	2.4	3.2	2.0
		Viruses	0.0	0.3	0.2
		WS1	0.0	0.1	0.0
		Zetaproteobacteria	0.1	0.0	0.1
		Zoopagomycota	0.0	0.0	0.0
	BS_A	Acetothermia	0.1	0.1	0.1
		Acidithiobacillia	0.1	0.0	0.1
		Acidobacteria	6.3	3.4	1.9
		Actinobacteria	3.5	2.9	3.7
		Aerophobetes	0.5	0.7	0.6
		Aigarchaeota	0.2	1.4	0.2
		Alphaproteobacteria	5.4	2.7	4.5
		Aminicenantes	1.6	2.0	1.4
		Annelida	0.0	0.0	0.0
		Apicomplexa	0.0	0.0	0.0
		Aquificae	0.2	0.3	0.3
		Armatimonadetes	0.4	0.3	0.3
		Arthropoda	0.0	0.0	0.0
		Ascomycota	0.1	0.1	0.1
		Atribacteria	0.5	0.5	0.4
		Bacillariophyta	0.0	0.0	0.0
		Bacteroidetes	2.8	3.6	6.5
		Balneolaeota	0.1	0.1	0.1
		Basidiomycota	0.0	0.1	0.0
		Betaproteobacteria	3.4	1.8	3.8
		Blastocladiomycota	0.0	0.0	0.0
		BRC1	0.4	0.3	0.5

		Caldiserica	0.1	0.1	0.1
		Caldithrixae	0.2	0.2	0.3
		Calescamantes	0.0	0.1	0.0
		Candidatus Atribacteria	0.2	0.2	0.2
		Candidatus Bipolaricaulota	0.0	0.1	0.0
		Candidatus Korarchaeota	0.1	0.4	0.1
		Candidatus Melainabacteria	0.0	0.0	0.0
		Candidatus Micrarchaeota	0.0	0.1	0.0
		Candidatus Microgenomates	0.1	0.0	0.0
		Candidatus Poribacteria	0.1	0.2	0.2
		Candidatus Saccharibacteria	0.0	0.0	0.0
		Chlamydiae	0.1	0.1	0.1
		Chlorobi	0.3	0.2	0.3
		Chloroflexi	11.1	8.1	6.6
		Chlorophyta	0.0	0.0	0.0
		Chordata	0.0	0.0	0.0
		Chrysiogenetes	0.0	0.0	0.0
		Chytridiomycota	0.0	0.0	0.0
		Cloacimonetes	0.2	0.3	0.3
		Cnidaria	0.0	0.0	0.0
		Coprothermobacterota	0.0	0.0	0.0
		Crenarchaeota	0.7	4.6	0.9
		Cyanobacteria	2.2	1.8	2.2
		Deferribacteres	0.1	0.1	0.1
		Deinococcus-Thermus	0.4	0.3	0.3
		Deltaproteobacteria	13.4	9.0	14.9
		Diapherotrites	0.0	0.2	0.1
		Dictyoglomi	0.1	0.2	0.1
		Elusimicrobia	0.0	0.1	0.1
		EM3	0.0	0.0	0.0
		Epsilonproteobacteria	0.1	0.1	0.2
		Euryarchaeota	17.9	21.8	13.5
		Fervidibacteria	0.2	0.2	0.1
		Fibrobacteres	0.1	0.1	0.2
		Firmicutes	10.1	13.6	11.6
		Fusobacteria	0.0	0.1	0.1
		Gammaproteobacteria	3.5	2.6	4.8
		Gemmatimonadetes	0.2	0.1	0.3
		Gracilibacteria	0.0	0.0	0.0
		Hydrogenedentes	0.3	0.3	0.4
		Hydrogenophilalia	0.0	0.0	0.0
		Ignavibacteriae	0.2	0.3	0.3
		Latescibacteria	0.5	0.7	1.1
		Lentisphaerae	0.1	0.1	0.2
		Marinimicrobia	0.1	0.2	0.2
		Microgenomates	0.1	0.1	0.1
		Microsporidia	0.0	0.0	0.0
		Mollusca	0.0	0.0	0.0
		Mucoromycota	0.0	0.0	0.0
		Nanoarchaeota	0.0	0.1	0.1
		Nematoda	0.0	0.0	0.0
		Nitrospinae	0.1	0.1	0.1
		Nitrospirae	1.2	0.9	1.0
		Oligoflexia	0.1	0.1	0.1
		Omnitrophica	0.2	0.3	0.5
		Parcubacteria	0.2	0.4	0.4

		PER	0.0	0.0	0.0
		Placozoa	0.0	0.0	0.0
		Planctomycetes	6.0	3.8	6.3
		Poribacteria	0.0	0.1	0.0
		Porifera	0.0	0.0	0.0
		Rhodothermaeota	0.0	0.0	0.1
		Spirochaetes	0.7	0.6	1.6
		Streptophyta	0.0	0.0	0.0
		Synergistetes	0.2	0.3	0.3
		Tenericutes	0.0	0.0	0.0
		Thaumarchaeota	0.5	3.1	0.6
		Thermodesulfobacteria	0.3	0.3	0.3
		Thermotogae	0.3	0.5	0.3
		unclassified Bacteria	0.0	0.0	0.1
		unclassified Eukaryota	0.1	0.1	0.1
		unclassified Proteobacteria	NA	0.0	0.0
		Verrucomicrobia	1.3	1.1	2.1
		Viruses	0.2	0.7	1.1
		WS1	0.1	0.0	0.0
		Zetaproteobacteria	0.0	0.0	0.0
		Zoopagomycota	0.0	0.0	0.0
captured	$SW_A \rightarrow TS_R$	Acetothermia	NA	0.0	0.0
		Acidithiobacillia	0.0	0.2	0.0
		Acidobacteria	0.6	0.2	0.3
		Actinobacteria	24.5	14.1	9.8
		Aerophobetes	0.0	0.0	0.0
		Aigarchaeota	0.0	NA	NA
		Alphaproteobacteria	15.0	16.0	12.2
		Aminicenantes	0.0	0.0	0.0
		Annelida	0.1	0.0	0.5
		Apicomplexa	0.3	0.1	0.2
		Aquificae	0.0	0.1	0.0
		Armatimonadetes	0.1	0.1	0.1
		Arthropoda	0.3	0.2	0.6
		Ascomycota	0.2	0.1	3.8
		Atribacteria	0.0	0.0	0.0
		Bacillariophyta	0.1	0.1	3.6
		Bacteroidetes	1.7	4.7	3.2
		Balneolaeota	0.0	0.0	0.0
		Basidiomycota	0.2	0.1	2.0
		Betaproteobacteria	20.7	21.5	13.3
		Blastocladiomycota	0.0	0.0	0.0
		BRC1	0.0	0.0	0.0
		Caldiserica	NA	0.0	0.0
		Caldithrixae	0.0	0.0	0.0
		Calescamantes	NA	0.0	0.0
		Candidatus Atribacteria	NA	0.0	NA
		Candidatus Korarchaeota	NA	NA	0.0
		Candidatus Melainabacteria	NA	0.1	0.0
		Candidatus Micrarchaeota	NA	0.0	NA
		Candidatus Microgenomates	0.0	0.0	0.0
		Candidatus Poribacteria	0.0	0.0	0.0
		Candidatus Saccharibacteria	0.0	0.0	NA
		Chlamydiae	0.0	0.1	0.0
		Chlorobi	0.1	0.2	0.1
		Chloroflexi	0.7	0.3	0.4

		Chlorophyta	0.1	0.1	0.1
		Chordata	0.3	0.1	0.6
		Chrysiogenetes	0.0	0.0	0.0
		Chytridiomycota	0.0	0.0	0.1
		Cloacimonetes	0.0	0.0	0.0
		Cnidaria	0.1	0.0	0.5
		Coprothermobacterota	0.0	NA	0.0
		Crenarchaeota	NA	0.0	0.0
		Cyanobacteria	6.3	4.4	19.1
		Deferribacteres	0.0	0.0	0.0
		Deinococcus-Thermus	0.1	0.1	0.2
		Deltaproteobacteria	2.2	1.8	1.0
		Diapherotrites	NA	0.0	NA
		Dictyoglomi	NA	0.0	0.0
		Elusimicrobia	0.0	0.0	0.0
		Epsilonproteobacteria	0.0	0.2	0.1
		Euryarchaeota	0.1	0.3	0.2
		Fervidibacteria	0.0	0.0	0.0
		Fibrobacteres	0.1	0.0	0.0
		Firmicutes	1.7	2.3	1.7
		Fusobacteria	0.0	0.0	0.2
		Gammaproteobacteria	5.1	8.3	3.9
		Gemmatimonadetes	0.1	0.0	0.9
		Hydrogenedentes	0.0	0.0	0.0
		Hydrogenophilalia	0.0	0.0	0.0
		Ignavibacteriae	0.0	0.0	0.0
		Latescibacteria	0.0	0.0	0.0
		Lentisphaerae	0.1	0.0	0.1
		Marinimicrobia	0.0	0.1	0.2
		Microgenomates	0.0	0.0	NA
		Microsporidia	0.0	0.0	0.0
		Mollusca	0.0	0.0	0.0
		Mucoromycota	0.0	0.0	0.0
		Nematoda	0.0	0.0	0.0
		Nitrospinae	0.0	0.0	0.0
		Nitrospirae	0.1	0.1	0.1
		Oligoflexia	0.2	0.2	0.1
		Omnitrophica	0.0	0.0	0.0
		Parcubacteria	0.0	0.0	0.0
		PER	NA	0.0	NA
		Placozoa	0.0	0.0	0.0
		Planctomycetes	7.9	1.7	5.4
		Poribacteria	0.0	0.0	NA
		Porifera	0.1	0.0	0.1
		Rhodothermaeota	0.0	0.0	0.0
		Spirochaetes	0.1	0.2	0.1
		Streptophyta	0.1	0.1	1.6
		Synergistetes	0.0	0.0	0.0
		Tenericutes	0.0	0.0	0.0
		Thaumarchaeota	0.0	0.0	0.0
		Thermodesulfobacteria	0.0	0.0	0.0
		Thermotogae	0.0	0.0	0.0
		unclassified Bacteria	NA	0.0	0.0
		unclassified Eukaryota	0.7	0.2	0.6
		Verrucomicrobia	4.2	2.4	5.1
		Viruses	5.1	18.7	7.2

		WS1	NA	NA	0.0
		Zetaproteobacteria	0.0	0.0	0.0
		Zoopagomycota	0.0	0.0	0.0
	$SW_A \rightarrow BS_R$	Acetothermia	0.0	NA	NA
		Acidithiobacillia	0.0	0.0	0.0
		Acidobacteria	0.3	0.1	0.1
		Actinobacteria	17.5	9.4	7.4
		Aerophobetes	0.0	0.0	NA
		Aigarchaeota	0.0	NA	NA
		Alphaproteobacteria	16.3	12.8	14.9
		Aminicenantes	0.0	NA	NA
		Annelida	0.0	0.0	0.0
		Apicomplexa	0.0	0.0	0.0
		Aquificae	0.0	0.1	0.0
		Armatimonadetes	0.1	0.0	0.0
		Arthropoda	0.3	0.0	0.1
		Ascomycota	0.2	0.1	0.1
		Atribacteria	0.0	NA	0.0
		Bacillariophyta	0.0	0.0	0.0
		Bacteroidetes	2.0	3.3	3.6
		Balneolaeota	0.0	0.0	0.0
		Basidiomycota	0.1	0.0	0.1
		Betaproteobacteria	20.5	12.1	13.4
		Blastocladiomycota	0.0	0.0	0.0
		BRC1	0.0	NA	0.0
		Caldiserica	NA	0.0	NA
		Caldithrixae	0.0	NA	0.1
		Candidatus Atribacteria	NA	NA	0.0
		Candidatus Melainabacteria	0.0	0.0	NA
		Candidatus Microgenomates	0.0	0.0	0.0
		Candidatus Poribacteria	0.0	0.0	0.0
		Candidatus Saccharibacteria	0.0	0.0	0.0
		Chlamydiae	0.0	0.2	0.0
		Chlorobi	0.1	0.2	0.0
		Chloroflexi	0.2	0.4	0.2
		Chlorophyta	0.1	0.1	0.1
		Chordata	0.1	0.0	0.0
		Chrysiogenetes	0.0	0.0	0.0
		Chytridiomycota	0.0	0.0	0.0
		Cloacimonetes	0.0	0.0	NA
		Cnidaria	0.0	0.0	0.0
		Crenarchaeota	0.0	NA	0.0
		Cyanobacteria	1.9	1.3	11.3
		Deferribacteres	0.0	NA	0.0
		Deinococcus-Thermus	0.4	0.1	0.3
		Deltaproteobacteria	1.2	1.5	1.2
		Diapherotrites	NA	0.0	NA
		Dictyoglomi	0.0	NA	NA
		Elusimicrobia	0.0	0.0	0.0
		Epsilonproteobacteria	0.2	0.3	0.1
		Euryarchaeota	0.1	0.6	0.2
		Fervidibacteria	0.0	0.0	0.0
		Fibrobacteres	0.0	0.0	0.0
		Firmicutes	2.4	2.3	2.4
		Fusobacteria	0.0	0.0	0.0
		Gammaproteobacteria	6.1	6.3	4.8

		Gemmatimonadetes	0.0	0.0	0.1
		Gracilibacteria	0.0	NA	NA
		Hydrogenedentes	0.0	NA	0.0
		Hydrogenophilalia	0.0	NA	0.0
		Ignavibacteriae	0.0	NA	0.0
		Latescibacteria	0.0	NA	0.0
		Lentisphaerae	0.0	0.0	0.0
		Marinimicrobia	0.0	0.0	0.0
		Microgenomates	0.0	0.0	NA
		Microsporidia	0.0	0.0	0.0
		Mollusca	0.0	0.0	0.0
		Mucoromycota	0.0	0.0	0.0
		Nanoarchaeota	0.0	NA	NA
		Nematoda	0.0	0.0	0.0
		Nitrospinae	0.0	NA	0.0
		Nitrospirae	0.1	0.1	0.0
		Oligoflexia	0.3	0.2	0.1
		Omnitrophica	0.0	NA	0.0
		Parcubacteria	0.0	0.0	0.0
		Placozoa	0.0	NA	0.0
		Planctomycetes	12.1	0.4	6.5
		Poribacteria	NA	0.0	NA
		Porifera	0.0	0.0	0.0
		Rhodothermaeota	0.0	0.0	0.0
		Spirochaetes	0.1	0.2	0.1
		Streptophyta	0.1	0.0	0.1
		Synergistetes	0.1	0.1	0.4
		Tenericutes	0.0	0.0	0.1
		Thaumarchaeota	0.0	0.0	0.0
		Thermodesulfobacteria	0.0	0.0	0.0
		Thermotogae	0.0	0.0	0.2
		unclassified Bacteria	0.0	0.0	NA
		unclassified Eukaryota	0.1	0.0	0.1
		Verrucomicrobia	1.5	0.9	2.8
		Viruses	15.2	46.6	28.9
		WS1	NA	NA	0.0
		Zetaproteobacteria	0.0	0.0	0.0
		Zoopagomycota	0.0	NA	0.0
	$TS_A \rightarrow BS_R$	Acetothermia	0.0	0.0	0.0
		Acidithiobacillia	0.1	0.0	0.1
		Acidobacteria	2.8	1.1	1.4
		Actinobacteria	5.2	2.2	3.4
		Aerophobetes	0.1	0.4	0.3
		Aigarchaeota	0.0	0.7	0.0
		Alphaproteobacteria	7.1	2.8	4.0
		Aminicenantes	2.5	0.8	3.9
		Annelida	0.0	0.0	0.0
		Apicomplexa	0.0	0.0	0.0
		Aquificae	0.2	0.2	0.1
		Armatimonadetes	0.1	0.2	0.1
		Arthropoda	0.0	0.0	0.0
		Ascomycota	0.0	0.0	0.0
		Atribacteria	0.1	0.3	0.2
		Bacillariophyta	0.0	0.0	0.0
		Bacteroidetes	1.6	3.5	2.3
		Balneolaeota	0.0	0.0	0.0

		Basidiomycota	0.0	0.0	0.0
		Betaproteobacteria	6.2	2.4	6.7
		Blastocladiomycota	0.0	0.0	0.0
		BRC1	0.1	0.3	0.1
		Caldiserica	0.0	0.0	0.0
		Caldithrixae	0.2	0.1	0.2
		Calescamantes	0.0	0.0	0.0
		Candidatus Atribacteria	0.1	0.1	0.1
		Candidatus Bipolaricaulota	0.0	0.0	0.0
		Candidatus Korarchaeota	0.0	0.1	0.0
		Candidatus Melainabacteria	0.0	0.0	0.0
		Candidatus Micrarchaeota	0.0	0.0	0.0
		Candidatus Microgenomates	0.0	0.0	0.0
		Candidatus Poribacteria	0.0	0.1	0.0
		Candidatus Saccharibacteria	0.0	0.0	0.0
		Chlamydiae	0.0	0.1	0.0
		Chlorobi	0.2	0.2	0.2
		Chloroflexi	2.7	5.3	3.4
		Chlorophyta	0.0	0.0	0.0
		Chordata	0.0	0.0	0.0
		Chrysiogenetes	0.0	0.0	0.0
		Chytridiomycota	0.0	0.0	0.0
		Cloacimonetes	0.1	0.2	0.1
		Cnidaria	0.0	0.0	0.0
		Coprothermobacterota	0.0	0.0	0.0
		Crenarchaeota	0.1	1.5	0.2
		Cyanobacteria	1.2	1.3	1.6
		Deferribacteres	0.2	0.1	0.1
		Deinococcus-Thermus	0.3	0.2	0.2
		Deltaproteobacteria	21.0	10.9	20.6
		Diapherotrites	0.0	0.1	0.0
		Dictyoglomi	0.0	0.1	0.1
		Elusimicrobia	0.0	0.1	0.0
		EM3	0.0	0.0	NA
		Epsilonproteobacteria	0.1	0.1	0.1
		Euryarchaeota	29.1	39.6	28.2
		Fervidibacteria	0.0	0.1	0.0
		Fibrobacteres	0.0	0.2	0.0
		Firmicutes	5.3	8.6	5.7
		Fusobacteria	0.0	0.0	0.0
		Gammaproteobacteria	5.2	2.9	6.9
		Gemmatimonadetes	0.4	0.1	0.7
		Gracilibacteria	0.0	0.0	0.0
		Hydrogenedentes	0.0	0.1	0.1
		Hydrogenophilalia	0.0	0.0	0.0
		Ignavibacteriae	0.2	0.1	0.2
		Latescibacteria	0.2	0.3	0.3
		Lentisphaerae	0.1	0.3	0.1
		Marinimicrobia	0.1	0.1	0.2
		Microgenomates	0.0	0.0	0.0
		Microsporidia	0.0	0.0	0.0
		Mollusca	0.0	0.0	0.0
		Mucoromycota	0.0	0.0	0.0
		Nanoarchaeota	0.0	0.0	0.0
		Nematoda	0.0	0.0	0.0
		Nitrospinae	0.1	0.1	0.1

		Nitrospirae	1.7	0.6	1.6
		Oligoflexia	0.1	0.1	0.1
		Omnitrophica	0.1	0.3	0.1
		Parcubacteria	0.0	0.0	0.0
		PER	0.0	0.0	0.0
		Placozoa	0.0	NA	0.0
		Planctomycetes	2.4	3.0	1.8
		Poribacteria	0.0	0.0	0.0
		Porifera	0.0	0.0	0.0
		Rhodothermaeota	0.0	0.0	0.0
		Spirochaetes	0.6	0.6	1.0
		Streptophyta	0.0	0.0	0.0
		Synergistetes	0.1	0.2	0.2
		Tenericutes	0.0	0.0	0.0
		Thaumarchaeota	0.1	1.4	0.1
		Thermodesulfobacteria	0.3	0.2	0.2
		Thermotogae	0.1	0.3	0.2
		unclassified Bacteria	0.0	0.0	0.1
		unclassified Eukaryota	0.0	0.0	0.0
		unclassified Proteobacteria	NA	0.0	NA
		Verrucomicrobia	0.8	1.9	0.9
		Viruses	0.0	2.8	0.9
		WS1	0.0	0.0	0.0
		Zetaproteobacteria	0.1	0.0	0.0
		Zoopagomycota	0.0	0.0	0.0
