# Supplementary material for: Sediment Metagenomes as Time Capsules of Lake Microbiomes
Source: mSphere. 2020 Nov 4;5(6):e00512-20. doi: 10.1128/mSphere.00512-20 (PMC7643826; doi:10.1128/mSphere.00512-20)
Supplement: TABLE S3 [file mSphere.00512-20-st003.pdf]

Table S3. Relative coverage of prevalent taxonomic orders (≥ 5% relative coverage in at least one metagenome) in the free and captured metagenomes of three lakes.

Metagenome type	Metagenome	Phylum	Order	Relative coverage (%)		
				Lac Paula	Eightmile Lake	Grand lac Touradi
free	SW_A	Actinobacteria	*Ca.* Nanopelagicales	12.9	20.0	35.2
			Corynebacteriales	3.8	0.6	0.9
		Alphaproteobacteria	Pelagibacterales	12.6	11.7	8.8
			Rhizobiales	3.2	2.0	2.0
			Sphingomonadales	0.7	1.1	0.4
		Betaproteobacteria	Burkholderiales	17.2	13.4	10.8
			Nitrosomonadales	1.6	1.0	1.8
		Cyanobacteria	Synechococcales	2.4	1.7	2.9
		Deltaproteobacteria	Desulfobacterales	0.1	0.1	0.1
			Desulfuromonadales	0.2	0.2	0.1
			Myxococcales	0.4	1.0	0.2
			Syntrophobacterales	0.1	0.1	0.0
		Euryarchaeota	Methanomicrobiales	0.0	0.0	0.0
		Firmicutes	Clostridiales	0.6	0.4	0.3
		Nitrospirae	Nitrospirales	0.1	0.1	0.1
		Planctomycetes	Planctomycetales	2.2	2.0	1.6
		Viruses	Caudovirales	1.7	0.9	0.2
	TS_A	Actinobacteria	*Ca.* Nanopelagicales	0.0	0.0	0.0
			Corynebacteriales	1.1	0.4	0.6
		Alphaproteobacteria	Pelagibacterales	0.0	0.0	0.0
			Rhizobiales	5.8	1.4	2.2
			Sphingomonadales	0.5	0.3	0.4
		Betaproteobacteria	Burkholderiales	7.2	2.2	6.3
			Nitrosomonadales	3.3	0.7	6.1
		Cyanobacteria	Synechococcales	0.6	0.7	0.8
		Deltaproteobacteria	Desulfobacterales	2.8	4.9	5.8
			Desulfuromonadales	4.8	2.3	3.0
			Myxococcales	6.8	1.6	3.2
			Syntrophobacterales	3.3	7.0	6.6
		Euryarchaeota	Methanomicrobiales	0.6	6.3	6.2
		Firmicutes	Clostridiales	2.6	4.1	2.3
		Nitrospirae	Nitrospirales	2.9	0.7	5.0
		Planctomycetes	Planctomycetales	2.4	3.2	1.4
		Viruses	Caudovirales	0.0	0.1	0.1
	BS_A	Actinobacteria	*Ca.* Nanopelagicales	0.0	0.0	0.0
			Corynebacteriales	0.5	0.4	0.7
		Alphaproteobacteria	Pelagibacterales	0.0	0.0	0.0
			Rhizobiales	3.7	1.4	2.4
			Sphingomonadales	0.3	0.3	0.4
		Betaproteobacteria	Burkholderiales	2.2	1.3	2.6
			Nitrosomonadales	0.6	0.3	0.6
		Cyanobacteria	Synechococcales	0.5	0.4	0.7
		Deltaproteobacteria	Desulfobacterales	2.6	2.2	3.6
			Desulfuromonadales	2.9	1.7	2.4
			Myxococcales	2.4	0.8	1.3
			Syntrophobacterales	3.3	2.3	4.8
		Euryarchaeota	Methanomicrobiales	12.4	4.3	6.9
		Firmicutes	Clostridiales	4.3	5.9	5.0

		Nitrospirae	Nitrospirales	1.1	0.8	0.9
		Planctomycetes	Planctomycetales	4.2	2.1	3.7
		Viruses	Caudovirales	0.1	0.3	0.5
captured	$SW_A \rightarrow TS_R$	Actinobacteria	*Ca.* Nanopelagicales	0.4	3.4	0.3
			Corynebacteriales	17.7	1.5	2.7
		Alphaproteobacteria	Pelagibacterales	0.1	2.0	0.2
			Rhizobiales	7.4	4.5	4.4
			Sphingomonadales	2.6	3.5	1.7
		Betaproteobacteria	Burkholderiales	16.6	15.2	10.7
			Nitrosomonadales	2.3	2.5	0.9
		Cyanobacteria	Synechococcales	4.4	2.8	13.3
		Deltaproteobacteria	Desulfobacterales	0.2	0.3	0.2
			Desulfuromonadales	0.2	0.1	0.1
			Myxococcales	1.2	0.8	0.3
			Syntrophobacterales	0.1	0.1	0.1
		Euryarchaeota	Methanomicrobiales	0.0	0.0	0.0
		Firmicutes	Clostridiales	0.6	0.7	0.6
		Nitrospirae	Nitrospirales	0.1	0.1	0.1
		Planctomycetes	Planctomycetales	7.7	1.5	5.2
		Viruses	Caudovirales	2.2	8.4	1.5
	$SW_A \rightarrow BS_R$	Actinobacteria	*Ca.* Nanopelagicales	0.0	0.1	0.1
			Corynebacteriales	15.1	1.7	5.2
		Alphaproteobacteria	Pelagibacterales	0.2	1.5	1.0
			Rhizobiales	6.4	5.6	4.6
			Sphingomonadales	5.3	1.8	5.9
		Betaproteobacteria	Burkholderiales	17.3	9.5	10.4
			Nitrosomonadales	2.1	1.3	2.4
		Cyanobacteria	Synechococcales	0.9	0.6	9.3
		Deltaproteobacteria	Desulfobacterales	0.2	0.3	0.4
			Desulfuromonadales	0.1	0.0	0.0
			Myxococcales	0.3	0.1	0.1
			Syntrophobacterales	0.1	0.0	0.1
		Euryarchaeota	Methanomicrobiales	0.0	0.0	0.0
		Firmicutes	Clostridiales	0.9	0.7	1.1
		Nitrospirae	Nitrospirales	0.1	0.1	0
		Planctomycetes	Planctomycetales	12.0	0.4	6.4
		Viruses	Caudovirales	5.0	14.2	6.6
	$TS_A \rightarrow BS_R$	Actinobacteria	*Ca.* Nanopelagicales	0.0	0.0	0.0
			Corynebacteriales	0.6	0.4	0.7
		Alphaproteobacteria	Pelagibacterales	0.0	0.0	0.0
			Rhizobiales	4.9	1.3	1.9
			Sphingomonadales	0.4	0.4	0.5
		Betaproteobacteria	Burkholderiales	3.4	1.7	4.1
			Nitrosomonadales	1.3	0.4	1.3
		Cyanobacteria	Synechococcales	0.4	0.3	0.7
		Deltaproteobacteria	Desulfobacterales	2.5	2.8	6.0
			Desulfuromonadales	5.5	2.0	2.5
			Myxococcales	6.5	0.8	1.8
			Syntrophobacterales	3.9	3.1	6.3
		Euryarchaeota	Methanomicrobiales	27.2	30.2	23.8
		Firmicutes	Clostridiales	2.3	4.0	2.6
		Nitrospirae	Nitrospirales	1.6	0.5	1.4
		Planctomycetes	Planctomycetales	1.9	2.0	1.0
		Viruses	Caudovirales	0.0	0.9	0.2
