# Supplementary material for: Sediment Metagenomes as Time Capsules of Lake Microbiomes
Source: mSphere. 2020 Nov 4;5(6):e00512-20. doi: 10.1128/mSphere.00512-20 (PMC7643826; doi:10.1128/mSphere.00512-20)
Supplement: FIG S3 [file mSphere.00512-20-sf003.pdf]

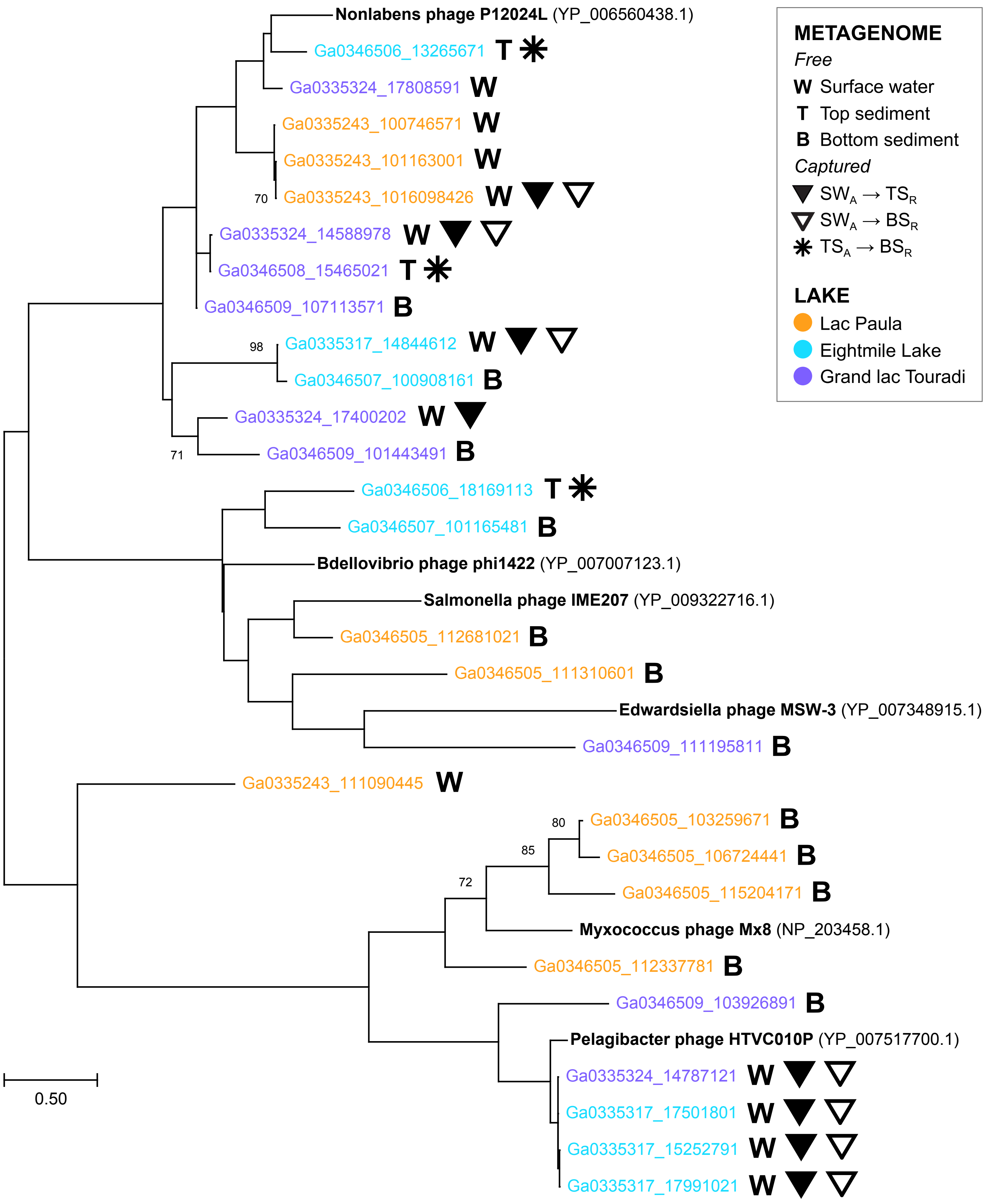
Nonlabens phage P12024L (YP_006560438.1)
Ga0346506_13265671 T
Ga0335324_17808591 W
Ga0335243_100746571 W
Ga0335243_101163001 W
70
Ga0335243_1016098426 W
Ga0335324_14588978 W
Ga0346508_15465021 T
Ga0346509_107113571 B
98
Ga0335317_14844612 W
Ga0346507_100908161 B
Ga0335324_17400202 W
71
Ga0346509_101443491 B
Ga0346506_18169113 T
Ga0346507_101165481 B
Bdellovibrio phage phi1422 (YP_007007123.1)
Salmonella phage IME207 (YP_009322716.1)
Ga0346505_112681021 B
Ga0346505_111310601 B
Edwardsiella phage MSW-3 (YP_007348915.1)
Ga0346509_111195811 B
Ga0335243_111090445 W
80
Ga0346505_103259671 B
85
Ga0346505_106724441 B
72
Ga0346505_115204171 B
Myxococcus phage Mx8 (NP_203458.1)
Ga0346505_112337781 B
Ga0346509_103926891 B
Pelagibacter phage HTVC010P (YP_007517700.1)
Ga0335324_14787121 W
Ga0335317_17501801 W
Ga0335317_15252791 W
Ga0335317_17991021 W
0.50
METAGENOME
Free
W Surface water
T Top sediment
B Bottom sediment
Captured
SW_A → TS_R
SW_A → BS_R
TS_A → BS_R
LAKE
Lac Paula
Eightmile Lake
Grand lac Touradi
