# Supplementary material for: Sediment Metagenomes as Time Capsules of Lake Microbiomes
Source: mSphere. 2020 Nov 4;5(6):e00512-20. doi: 10.1128/mSphere.00512-20 (PMC7643826; doi:10.1128/mSphere.00512-20)
Supplement: TABLE S4 [file mSphere.00512-20-st004.pdf]

Table S4. Accession information for Betaproteobacteria reference genomes downloaded from IMG.

IMG Genome ID	Genome Name / Sample Name	GOLD Analysis Project ID	GOLD Analysis Project Type	GOLD Study ID	Add Date
2503982034	Polynucleobacter necessarius STIR1	Ga0025122	Genome Analysis (Isolate)	Gs0019822	2010-12-22
2517572181	Polynucleobacter sp. MWH-UH21B	Ga0011490	Genome Analysis (Isolate)	Gs0019823	2012-10-31
2574179738	Polynucleobacter sphagniphilus MWH-Weng1-1	Ga0048563	Genome Analysis (Isolate)	Gs0019823	2014-06-12
2590828818	Polynucleobacter hirudinilacicola MWH-EgelM1-30-B4	Ga0059496	Genome Analysis (Isolate)	Gs0019823	2014-09-29
2590828843	Polynucleobacter campilacus MWH-Feld-100	Ga0059497	Genome Analysis (Isolate)	Gs0019823	2014-09-29
2596583565	Polynucleobacter sp. FNE-F8 bin_6_1_PnecC	Ga0061027	Metagenome-Assembled Genome	Gs0090293	2014-12-02
2634166443	Polynucleobacter duraquae MWH-MoK4	Ga0077867	Genome Analysis (Isolate)	Gs0019823	2015-10-08
2636415981	Polynucleobacter asymbioticus Recht-Kol-4	Ga0082235	Genome Analysis (Isolate)	Gs0113789	2015-10-20
2636416036	Polynucleobacter asymbioticus Tro8-F10W22	Ga0082243	Genome Analysis (Isolate)	Gs0113789	2015-10-20
2636416061	Polynucleobacter asymbioticus Tro-7-1-4	Ga0098027	Genome Analysis (Isolate)	Gs0113789	2015-10-20
2636416062	Polynucleobacter asymbioticus Recht-Kol-B GCA_001874545.1	Ga0098029	Genome Analysis (Isolate)	Gs0113789	2015-10-20
2639762512	Polynucleobacter asymbioticus P1-4-10KL	Ga0099456	Genome Analysis (Isolate)	Gs0113789	2015-11-06
2639762514	Polynucleobacter asymbioticus Recht-1	Ga0099459	Genome Analysis (Isolate)	Gs0113789	2015-11-06
2639762515	Polynucleobacter asymbioticus Tro-8-2-9	Ga0099460	Genome Analysis (Isolate)	Gs0113789	2015-11-06
2642422500	Polynucleobacter sp. MWH-Tro-8-2-5GR	Ga0062610	Genome Analysis (Isolate)	Gs0113858	2015-11-24
2642422572	Polynucleobacter TBE6_bin-23_Pnec	Ga0100165	Metagenome-Assembled Genome	Gs0090293	2015-11-24
2642422578	Polynucleobacter TBE6_bin-10_Pnec	Ga0100171	Metagenome-Assembled Genome	Gs0090293	2015-11-24
2675903126	Polynucleobacter aenigmaticus MWH-K35W1 reannotation	Ga0129263	Genome Analysis (Isolate)	Gs0019823	2016-06-15
2684622883	Limnohabitans sp. Rim47 finished	Ga0132226	Genome Analysis (Isolate)	Gs0114543	2016-07-12
2687453598	Polynucleobacter wuianus QLW-P1FAT50C-4	Ga0131698	Genome Analysis (Isolate)	Gs0019823	2016-07-27
2690315905	Limnohabitans sp. Rim47	Ga0132431	Genome Analysis (Isolate)	Gs0120468	2016-08-11
2708742530	Polynucleobacter sp. VK13	Ga0139086	Genome Analysis (Isolate)	Gs0110196	2016-12-09
2710264786	Polynucleobacter victoriensis MWH-VicM1	Ga0139087	Genome Analysis (Isolate)	Gs0110196	2016-12-19
2710724120	Polynucleobacter meluiroseus AP-Melu-1000-B4	Ga0139085	Genome Analysis (Isolate)	Gs0110196	2017-01-10
2710724123	Polynucleobacter sp. UB-Domo-W1	Ga0139088	Genome Analysis (Isolate)	Gs0110196	2017-01-10
2721755102	Polynucleobacter sp. GWA2_45_21	Ga0154321	Metagenome-Assembled Genome	Gs0053054	2017-03-23
2724679690	Limnohabitans sp. bin 1	Ga0182879	Metagenome-Assembled Genome	Gs0128842	2017-04-11
2737471665	Polynucleobacter sp. JGI_MCM14TBH076 (contamination screened)	Ga0191053	Single Cell Analysis (screened)	Gs0129090	2017-06-16
2737471704	Limnohabitans sp. JGI_MCM14ME275 (contamination screened)	Ga0191061	Single Cell Analysis (screened)	Gs0129090	2017-06-16
2737472025	Polynucleobacter sp. JGI_MCM14ME033 (contamination screened)	Ga0191041	Single Cell Analysis (screened)	Gs0129090	2017-06-16
2739367523	Limnohabitans sp. JGI_MCM14ME184 (contamination screened)	Ga0191097	Single Cell Analysis (screened)	Gs0129090	2017-07-17
2739367531	Limnohabitans sp. JGI_MCM14ME254 (contamination screened)	Ga0191089	Single Cell Analysis (screened)	Gs0129090	2017-07-17

2739367535	Polynucleobacter sp. JGI_MCM14ME153 (contamination screened)	Ga0191081	Single Cell Analysis (screened)	Gs0129090	2017-07-17
2739367551	Polynucleobacter sp. JGI_MCM14TBH079 (contamination screened)	Ga0191109	Single Cell Analysis (screened)	Gs0129090	2017-07-17
2739367573	Polynucleobacter sp. JGI_MCM14TBH005 (contamination screened)	Ga0191115	Single Cell Analysis (screened)	Gs0129090	2017-07-17
2739367576	Polynucleobacter sp. JGI_MCM14TBH017 (contamination screened)	Ga0191117	Single Cell Analysis (screened)	Gs0129090	2017-07-17
2739367578	Limnohabitans sp. JGI_MCM14ME171 (contamination screened)	Ga0191127	Single Cell Analysis (screened)	Gs0129090	2017-07-17
2739367609	Polynucleobacter sp. JGI_MCM14TBH008 (contamination screened)	Ga0191165	Single Cell Analysis (screened)	Gs0129090	2017-07-17
2739367611	Limnohabitans sp. JGI_MCM14ME290 (contamination screened)	Ga0191207	Single Cell Analysis (screened)	Gs0129090	2017-07-17
2739367623	Polynucleobacter sp. JGI_MCM14TBH064 (contamination screened)	Ga0191197	Single Cell Analysis (screened)	Gs0129090	2017-07-17
2757320395	Limnohabitans sp. bin L8r	Ga0224457	Metagenome-Assembled Genome	Gs0110155	2017-11-13
2757320396	Limnohabitans sp. bin L5r	Ga0224458	Metagenome-Assembled Genome	Gs0110155	2017-11-13
2757320397	Limnohabitans sp. bin L6r	Ga0224459	Metagenome-Assembled Genome	Gs0110155	2017-11-13
2757320398	Limnohabitans sp. bin L7r	Ga0224460	Metagenome-Assembled Genome	Gs0110155	2017-11-13
2757320399	Limnohabitans sp. bin L3r	Ga0224461	Metagenome-Assembled Genome	Gs0110155	2017-11-13
2757320400	Limnohabitans sp. bin L1r	Ga0224462	Metagenome-Assembled Genome	Gs0110155	2017-11-13
2757320401	Limnohabitans sp. bin L02	Ga0224463	Metagenome-Assembled Genome	Gs0110155	2017-11-13
2757320402	Limnohabitans sp. bin L01	Ga0224464	Metagenome-Assembled Genome	Gs0110155	2017-11-13
2757320403	Limnohabitans sp. bin L03	Ga0224465	Metagenome-Assembled Genome	Gs0110155	2017-11-13
2757320404	Limnohabitans sp. bin L2r	Ga0224466	Metagenome-Assembled Genome	Gs0110155	2017-11-13
2770939593	Polynucleobacter paneuropaeus MWH-Creno-4B4 (version 2)	Ga0256620	Genome Analysis (Isolate)	Gs0110283	2018-02-21
2770939597	Polynucleobacter paneuropaeus FUKU-NW11 (version 2)	Ga0256624	Genome Analysis (Isolate)	Gs0114446	2018-02-21
2770939599	Polynucleobacter paneuropaeus MWH-CNW20-3 (version 2)	Ga0256626	Genome Analysis (Isolate)	Gs0114446	2018-02-21
2773857958	Limnohabitans sp. Rim47	Ga0154067	Genome Analysis (Isolate)	Gs0017198	2018-03-28
2799112297	Polynucleobacter necessarius PPGSP1	Ga0310996	Genome Analysis (Isolate)	Gs0135751	2018-09-26
2799112298	Polynucleobacter necessarius PPGSP4	Ga0311023	Genome Analysis (Isolate)	Gs0135751	2018-09-26
2799112299	Polynucleobacter necessarius PPGSP2	Ga0310993	Genome Analysis (Isolate)	Gs0135751	2018-09-26
2799112300	Polynucleobacter necessarius PPGSP5	Ga0310992	Genome Analysis (Isolate)	Gs0135751	2018-09-26
2799112301	Polynucleobacter necessarius PPGSP6	Ga0310997	Genome Analysis (Isolate)	Gs0135751	2018-09-26
2799112302	Polynucleobacter necessarius PPGSP7	Ga0310994	Genome Analysis (Isolate)	Gs0135751	2018-09-26
2799112303	Polynucleobacter necessarius PPGSP3	Ga0310995	Genome Analysis (Isolate)	Gs0135751	2018-09-26
2799112304	Polynucleobacter necessarius PPGSP8	Ga0310999	Genome Analysis (Isolate)	Gs0135751	2018-09-26
2834088864	Limnohabitans parvus II-B4	Ga0337021	Genome Analysis (Isolate)	Gs0139103	2019-08-21
2841307470	Limnohabitans sp. Rim11	Ga0392967	Genome Analysis (Isolate)	Gs0114543	2019-10-04

2841310320	Limnohabitans sp. 2KL-3	Ga0392968	Genome Analysis (Isolate)	Gs0114543	2019-10-04
2844342787	Polynucleobacter asymbioticus P1-Kol8	Ga0248401	Genome Analysis (Isolate)	Gs0113789	2019-11-12
2876966433	Polynucleobacter paneuropaeus MWH-UK1W16	Ga0170652	Genome Analysis (Isolate)	Gs0019823	2020-06-17
2876968418	Polynucleobacter sp. QLW-P1DATA-2	Ga0157492	Genome Analysis (Isolate)	Gs0019823	2020-06-17
2879185622	Polynucleobacter sp. UK-Long2-W17	Ga0439329	Genome Analysis (Isolate)	Gs0110283	2020-07-07
