# Supplementary material for: Sediment Metagenomes as Time Capsules of Lake Microbiomes
Source: mSphere. 2020 Nov 4;5(6):e00512-20. doi: 10.1128/mSphere.00512-20 (PMC7643826; doi:10.1128/mSphere.00512-20)
Supplement: TABLE S5 [file mSphere.00512-20-st005.pdf]

Table S5. GOLD Analysis Project IDs and links for annotated metagenome assemblies in this study.

Lake	Metagenome	GOLD Analysis Project	URL
Lac Paula	SW_A	Ga0335243	https://gold.jgi.doe.gov/analysis_project?id=Ga0335243
	TS_A	Ga0346504	https://gold.jgi.doe.gov/analysis_project?id=Ga0346504
	BS_A	Ga0346505	https://gold.jgi.doe.gov/analysis_project?id=Ga0346505
Eightmile Lake	SW_A	Ga0335317	https://gold.jgi.doe.gov/analysis_project?id=Ga0335317
	TS_A	Ga0346506	https://gold.jgi.doe.gov/analysis_project?id=Ga0346506
	BS_A	Ga0346507	https://gold.jgi.doe.gov/analysis_project?id=Ga0346507
Grand lac Touradi	SW_A	Ga0335324	https://gold.jgi.doe.gov/analysis_project?id=Ga0335324
	TS_A	Ga0346508	https://gold.jgi.doe.gov/analysis_project?id=Ga0346508
	BS_A	Ga0346509	https://gold.jgi.doe.gov/analysis_project?id=Ga0346509
